# Confocal Microscopy-Based Estimation of Parameters for Computational Modeling of Electrical Conduction in the Normal and Infarcted Heart

**DOI:** 10.3389/fphys.2018.00239

**Published:** 2018-04-04

**Authors:** Joachim Greiner, Aparna C. Sankarankutty, Gunnar Seemann, Thomas Seidel, Frank B. Sachse

**Affiliations:** ^1^Nora Eccles Harrison Cardiovascular Research and Training Institute, University of Utah, Salt Lake City, UT, United States; ^2^Institute of Biomedical Engineering, Karlsruhe Institute of Technology, Karlsruhe, Germany; ^3^Bioengineering Department, University of Utah, Salt Lake City, UT, United States; ^4^Institute for Experimental Cardiovascular Medicine, University Heart Center, Medical Center University of Freiburg, Freiburg, Germany; ^5^Faculty of Medicine, Albert Ludwigs University of Freiburg, Freiburg, Germany; ^6^Institute for Cellular and Molecular Physiology, University of Erlangen-Nuremberg, Erlangen, Germany

**Keywords:** cardiac modeling, cardiac tissue, myocardial infarction, tissue conductivity, electrical conduction

## Abstract

Computational modeling is an important tool to advance our knowledge on cardiac diseases and their underlying mechanisms. Computational models of conduction in cardiac tissues require identification of parameters. Our knowledge on these parameters is limited, especially for diseased tissues. Here, we assessed and quantified parameters for computational modeling of conduction in cardiac tissues. We used a rabbit model of myocardial infarction (MI) and an imaging-based approach to derive the parameters. Left ventricular tissue samples were obtained from fixed control hearts (animals: 5) and infarcted hearts (animals: 6) within 200 μm (region 1), 250–750 μm (region 2) and 1,000–1,250 μm (region 3) of the MI border. We assessed extracellular space, fibroblasts, smooth muscle cells, nuclei and gap junctions by a multi-label staining protocol. With confocal microscopy we acquired three-dimensional (3D) image stacks with a voxel size of 200 × 200 × 200 nm. Image segmentation yielded 3D reconstructions of tissue microstructure, which were used to numerically derive extracellular conductivity tensors. Volume fractions of myocyte, extracellular, interlaminar cleft, vessel and fibroblast domains in control were (in %) 65.03 ± 3.60, 24.68 ± 3.05, 3.95 ± 4.84, 7.71 ± 2.15, and 2.48 ± 1.11, respectively. Volume fractions in regions 1 and 2 were different for myocyte, myofibroblast, vessel, and extracellular domains. Fibrosis, defined as increase in fibrotic tissue constituents, was (in %) 21.21 ± 1.73, 16.90 ± 9.86, and 3.58 ± 8.64 in MI regions 1, 2, and 3, respectively. For control tissues, image-based computation of longitudinal, transverse and normal extracellular conductivity yielded (in S/m) 0.36 ± 0.11, 0.17 ± 0.07, and 0.1 ± 0.06, respectively. *Conductivities were markedly increased in regions 1 (*+*75*, +*171, and* +*100%), 2 (*+*53*, +*165, and* +*80%), and 3 (*+*42*, +*141, and* +*60%)*. Volume fractions of the extracellular space including interlaminar clefts strongly correlated with conductivities in control and MI hearts. Our study provides novel quantitative data for computational modeling of conduction in normal and MI hearts. Notably, our study introduces comprehensive statistical information on tissue composition and extracellular conductivities on a microscopic scale in the MI border zone. We suggest that the presented data fill a significant gap in modeling parameters and extend our foundation for computational modeling of cardiac conduction.

## Introduction

Cardiac diseases, such as myocardial infarction (MI), are often associated with an increased risk of arrhythmia, which results from remodeling of cellular electrophysiology and tissue electrical properties. Computational modeling is a major research approach to study underlying mechanisms and effects of cardiac diseases, as well as cardiac physiology (Sachse, 2004; Moreno and Clancy, 2009; Clayton et al., 2011).

Established models of cardiac electrical conduction include the monodomain and bidomain models. More recently, multidomain models of cardiac conduction were introduced (Sachse et al., 2009). Computational simulations with these models commonly require discretization of spatial domains with finite element or finite difference methods. Models of cellular electrophysiology are assigned to elements or nodes of the computational mesh of conduction models. These models of cellular electrophysiology describe voltages across the cell membrane *V*_*m*_ and associated membrane currents. Commonly, only models of myocyte electrophysiology are considered, because myocytes occupy most of the volume in cardiac tissues and with some exceptions, the contribution of other cells to conduction is thought to be marginal throughout the normal heart. Models of myocyte electrophysiology have been developed for various species and anatomical regions (Lloyd et al., 2008; Fink et al., 2011). However, various experimental findings suggest that non-myocytes, in particular, fibroblasts, myofibroblasts and macrophages, contribute to cardiac conduction and arrhythmia (Gaudesius et al., 2003; Miragoli et al., 2006; Zlochiver et al., 2008; Quinn et al., 2016; Hulsmans et al., 2017). Electrophysiological models of these cells have been developed and their effects on conduction can be simulated using multi-domain models.

Beyond cell models, intracellular electrical conductivities σ_*i*_ and, for the bidomain and multidomain models, extracellular electrical conductivities σ_*e*_ are assigned to elements or nodes in a mesh for modeling of cardiac conduction. These conductivities describe electrical properties resulting from tissue microstructure including the distribution and shape of cells, their intercellular coupling and the distribution of extracellular space. Commonly the conductivities are described by tensors of 2nd order to account for anisotropy, i.e., a strong directional dependence of conductivity characteristic for cardiac tissues. Interestingly, only a small number of studies have reported on anisotropic intra- and extracellular conductivities of cardiac tissues. Noteworthy are in particular studies analyzing electrical measurements on right ventricular (RV) trabecular bundles of calf (Clerc, 1976) and left ventricular (LV) subepicardial myocardium of canine (Roberts et al., 1979; Roberts and Scher, 1982). These studies yielded bidomain conductivities distinguishing between longitudinal and transverse conductivities of the extracellular and intracellular space, which relate to longitudinal and transverse orientation of the local myocytes. Recent work aims at establishing bidomain conductivities accounting for longitudinal and transverse orientation of myocytes as well as the orientation of myocyte sheets (Legrice et al., 1995; Hooks et al., 2002, 2007; Johnston, 2016). These sheets have been identified using optical and electron microscopy in LV and RV tissues of canine. The myocyte sheets are separated by interlaminar clefts. Our recent study revealed the presence of sheets in LV myocardium of rabbit and in a rabbit model of myocardial infarction (MI) (Seidel et al., 2016).

Difficulties in implementing simulations with computational models are related to identifying conductivities and other crucial parameter values for a specific tissue type and species. Selection of conductivities and many other modeling parameters for diseased cardiac tissues is even more difficult, because we do not have a similar solid foundation of measurement studies as for control tissues. Bidomain conductivities for modeling of conduction in MI have not been measured, but are estimated based on simple assumptions or indirect measurements. Currently, we have only a vague understanding of how disease-associated remodeling of tissue microstructure influences conductivities. However, new research tools, in particular, advanced microscopy methods, allow us now to comprehensively quantify remodeling in diseased cardiac tissues. In case of MI, tissue remodeling goes beyond the actual infarct scar and affects microstructure of the adjacent myocardium. Major microstructural remodeling of the infarct border zone is related to fibrosis, i.e., the increase of extracellular matrix proteins as well as volume fractions of extracellular space, fibroblasts, and myofibroblasts (Krenning et al., 2010). Fibrosis in the border zone is accompanied by reduction of volume fraction of myocytes, their coupling by gap junctions, and myocyte hypertrophy (Luke and Saffitz, 1991; Yao et al., 2003).

Here, we investigated how tissue microstructure and its variability in the normal and MI heart are reflected in parameters for conduction models. We applied an animal model of MI and a microscopic imaging-based approach to derive modeling parameters. A particular focus was on computational estimation and statistical prediction of electrical conductivities of the extracellular domain. We assessed the effect of extracellular space and interlaminar clefts on electrical conductivities.

## Methods

### Animal models

All animal experiments were approved by the Institutional Animal Care and Use Committee (IACUC) of the University of Utah. Studies were performed on New Zealand White rabbits with a weight of 2.5–3 kg. Five animals served as control. We induced LV MI in six animals by ligation of a coronary artery as previously described (Hu et al., 2010). Hearts were harvested after 3 weeks. We visually confirmed success of the MI model. Infarct scars were present in the apical region of the free left-ventricular wall in all MI animals.

### Preparation and labeling of samples for imaging

Hearts were rapidly excised and retrogradely perfused with 2.5% paraformaldehyde solution for 10 min. Transmural biopsies of 5 mm diameter oriented normal to the epicardial surface were obtained from the lateral LV free wall of control hearts. Similar biopsies from MI hearts were taken by placing the center of the biopsy punch on the epicardial or endocardial MI border zone. Biopsies were incubated in 30% sucrose solution for 2–3 h, frozen in optimal cutting temperature compound (Sakura Finetek Europe B.V., Alphen aan den Rijn, Netherlands) and then cryosectioned into slices with a thickness of 100 μm. Subepicardial and midwall slices were washed three times in phosphate-buffered saline (PBS). Primary antibodies ab11369 (Abcam, Cambridge, UK), A5228 and V6630 (Sigma-Aldrich, St. Louis, MO, USA) were applied at a concentration of 1:200 in blocking solution to bind to the proteins connexin 43 (Cx43), alpha smooth muscle actin (α-SMA) and vimentin, respectively. Slices were incubated for at least 8 h on a rocker at room temperature. After washing with PBS, corresponding goat anti-mouse secondary antibodies A21044 (conjugated to AF 594, Thermo Fisher Scientific, Waltham, MA, USA), A21137 (conjugated to AF 555, Thermo Fisher Scientific), and A21240 (conjugated to AF 647, Thermo Fisher Scientific) were applied at 1:200 in blocking solution together with DAPI (D3571, Thermo Fisher Scientific) at 3 μg/ml to label the nuclei. After incubation of the slices for at least 6 h on rocker at room temperature, they were washed with PBS and incubated with wheat germ agglutinin (WGA) conjugated to CF488A (Biotium Inc., Fremont, CA, USA) at a concentration of 40 μg/mL in PBS for at least 4 h to label the glycocalyx and extracellular matrix proteins. Slices were then washed in PBS. All used antibodies and fluorescent markers are listed in Table S1. We used a compression-free mounting method (Seidel et al., 2016). In short, slices were mounted on a coverslip using Fluoromount-G (#17984-25, Electron Microscopy Science, Hatfield, PA, USA) and stored in a humidity-controlled box. After drying for 24 h, samples were protected from desiccation by coating with varnish.

### Imaging using scanning confocal microscopy

Prepared coverslips were imaged using a laser scanning confocal microscope Leica TCS SP8 with a 40x oil immersion objective at a resolution of 1,024 × 1,024 pixels and an image size of 204 × 204 μm.

In tissue samples from MI hearts, the infarct scar was identified as fibrotic tissue without any myocytes. We took images within 200 μm (region 1), within 250–750 μm (region 2) and within 1,000–1,250 μm (region 3) of the scar border. Only image stacks without labeling or imaging artifacts as well as without presence of larger vessels were selected for further processing. A rotation of the field of view was applied before acquisition to yield a uniform myocyte orientation parallel to y axis. Three-dimensional image stacks were acquired in 3 sequences comprising 2, 2, and 1 channel(s) each, to collect signals from DAPI, Cx43, WGA, α-SMA, and vimentin, which were excited by lasers of wavelengths 405, 594, 488, 561, and 633 nm, respectively. Image stacks of 200–300 images covered the depth of each sample with a spacing of 200 nm between the images. Beyond individual image stacks, we also acquired 3D tile scan images covering larger regions of interest.

To compensate for decreasing signal intensity at increased depths within the tissue, excitation compensation was applied by linearly increasing the laser power with depth. Imaging software used was LAS X (version 1.1.0 and higher, Leica).

### Image preprocessing and segmentation

We described our methods and software tools for preprocessing and segmentation of three-dimensional images of cardiac tissues previously (Seidel et al., 2016). In short, Gaussian and mean filters were used to reduce noise in the acquired image stacks. We performed an attenuation correction to account for depth-dependent signal attenuation and the linear increase of laser power in our imaging protocol. A deconvolution method based on the Richardson-Lucy algorithm with previously measured point spread function was used to reduce blurring.

We produced signal intensity profiles of the five channels of a representative 3D tile scan spanning from the infarct scar to region 3. Signal intensity was averaged along the depth and width of the image stack. Normalized values of mean intensities over every 100 μm was plotted against the distance from the scar border.

For 3D reconstruction, a semi-automatic segmentation approach based on multiple watersheds was used to segment myocyte, interlaminar cleft, and vessel domains (Seidel et al., 2013). Occasionally, when the WGA signal was insufficient for accurate watershed segmentation, manual separation of segments was performed using the volume segmentation and processing tool Seg3D (SCI, 2015). After segmentation of myocytes, capillaries were identified and dilated to include vessel walls, containing endothelial, smooth muscle cells, and pericytes. Assuming that beyond myocytes and vessel-associated cells, only fibroblasts and myofibroblasts exhibit a significant cell population in the studied samples, we segmented the fibroblast and myofibroblast domain using histogram-based thresholding on DAPI, vimentin and α-SMA images as previously described (Seidel et al., 2016). Briefly, nuclei not associated with myocytes or proximal to vessels were attributed to the fibroblast or myofibroblast domain, dependent on proximity to vimentin and α-SMA-positive regions, respectively. Fibroblasts were defined as exclusively vimentin-positive cells not proximal to vessels. Myofibroblasts were defined as vimentin- and α-SMA-positive cells not proximal to vessels.

Extracellular space was defined by the residual space after the exclusion of segmented myocytes, vessels, fibroblasts, and myofibroblasts. We also measured the volume ratio of interlaminar clefts *V*_*cleft*_, which are a component of the extracellular domain (Legrice et al., 1995). Interlaminar clefts were identified as continuous regions in the extracellular space with low WGA signal due to reduced collagen density. Segmented image stacks were sequentially combined to generate comprehensive 3D reconstructions of tissue microstructure.

We calculated volume fractions of the myocyte *V*_*myo*_, fibroblast *V*_*fibro*_, myofibroblast *V*_*myofibro*_, extracellular *V*_*e*_ (including *V*_*cleft*_), and vessel domain *V*_*vessel*_ for control tissue and for regions 1–3 from the 3D reconstructions.

We defined fibrosis as the excess of fibroblasts and myofibroblasts as well as extracellular space excluding interlaminar clefts vs. control tissue. Accordingly, we calculated the degree of fibrosis in region x, where x is 1–3, from volume fractions of relevant tissue constituents as:

(1) $$\begin{array}{l} Fibrosis_{region\;x}=(V_{region\;x,e}-V_{region\;x,cleft}+V_{region\;x,fibro}\\ \;+V_{region\;x,myofibro})-(V_{control,e}-V_{control,cleft}\\ \;+V_{control,fibro}+V_{control,myofibro}) \end{array}$$

### Image-based computation of extracellular conductivities

Similar as in our prior work (Schwab et al., 2013), the 3D reconstructions of tissue microstructure were used to construct conductivity models, assuming that only the extracellular space contributes to extracellular current flow. The segmented extracellular space was further refined by the addition of a thresholded WGA image (mode + 1SD) inside the myocyte domain to conservatively preserve the interface of the extracellular space to the myocytes. We checked for alignment of tissue microstructure with the coordinate system of the conductivity model using principal component analysis of the 10 largest myocytes and the largest cleft of each stack. Stacks with fiber orientation or cleft orientation deviating from this definition were appropriately rotated. After rotation, the stacks were cropped to avoid undefined regions.

In order to derive the homogenized conductivities, an electrical field was applied to the extracellular conductivity model. The electrical field was incorporated in the model with Dirichlet boundary conditions. The field was applied in three axes of the coordinate system: longitudinal, i.e., along the myocyte fiber orientation, transverse, i.e., normal to the fiber orientation and in cleft plane, and normal, i.e., normal to both the fiber orientation and cleft plane. We denoted these directions with the subscripts *l*, *t*, and *n* respectively. We assumed a conductivity value of 2 S/m for the extracellular space (Foster and Schwan, 1989). The extracellular potential distribution ϕ_*e*_ was obtained by solving the resulting homogenous Laplace equation for a given extracellular conductivity distribution σ_*e*_:

$$\begin{array}{l} \nabla \cdot \left(\sigma_{e}\nabla \phi_{e}\right)=\;0 \end{array}$$

Surfaces of the extracellular conductivity model were assigned no-flux Neumann boundary conditions. From the potential distribution of the extracellular domain, the mean directional current densities *J*_*e,l*_, *J*_*e,t*_, and *J*_*e,n*_ were calculated. The current densities were used to derive the homogenized extracellular conductivities in the defined directions:

$$\begin{array}{l} \sigma_{e,x}=\;\frac{J_{e,x}}{E_{e,x}} \end{array}$$

A central finite difference scheme was used to numerically discretize the simulation domain. The resulting linear systems of equations were solved using the scientific computing library PETSc (Balay et al., 1997). The external library, Hypre, provided the parallel algebraic multigrid preconditioner BoomerAMG (Falgout and Yang, 2002), which was used in combination with PETSc's implementation of the iterative flexible generalized minimal residual (FGMRES) method. Algorithm configuration for the algebraic multigrid was optimized using the software package SMAC (Hutter et al., 2011) on a small test case. This yielded a V-cycle multigrid with a threshold for strong coupling of 0.12, HMIS-coarsening with one level of aggressive coarsening, extended+i interpolation, Schwartz-type smoothers and a CF-relaxation scheme. The iterative solver was stopped when the residual norm of the original linear system was <10^−10^. For post-processing and visualization, we used MATLAB (version 2016b and higher, MathWorks, Inc., Natick, MA, USA) and Paraview (version 5.4 and higher, Kitware, Clifton Park, New York, USA) respectively.

### Numerical verification

To verify our numerical method of estimating a homogenized conductivity, we performed a series of tests. We chose non-intersecting rectangular blocks as the testing geometry, as the derivation of the analytical solution is straightforward. The blocks were assigned to the extracellular conductivity model, whereas the remainder of the simulation domain was set non-conductive. For these geometries, a conductivity estimation was performed in each direction as described in section Image-Based Computation of Extracellular Conductivities. For varying geometries and simulation domains in the order of 40 × 40 × 40 μm and a spatial discretization of 200 nm, the error of the estimated conductivity was of the order 10^−8^.

### Statistical analyses

Statistical data are presented as mean ± standard deviation (SD). For each parameter tested, regions 1–3 and control were compared using the Holm-Bonferroni method for correction of multiple comparison. *P*-values were calculated using unpaired, two-tailed *t*-tests. A significance level (alpha) of 0.05 was used. Statistical relationships between tissue constituents and conductivities were analyzed using simple and multiple linear regression. The goodness of fit was measured by the coefficient of determination (*R*^2^). All statistical analyses were performed in MATLAB.

## Results

### Remodeling of myocardium in MI animals

**Figure 1:**
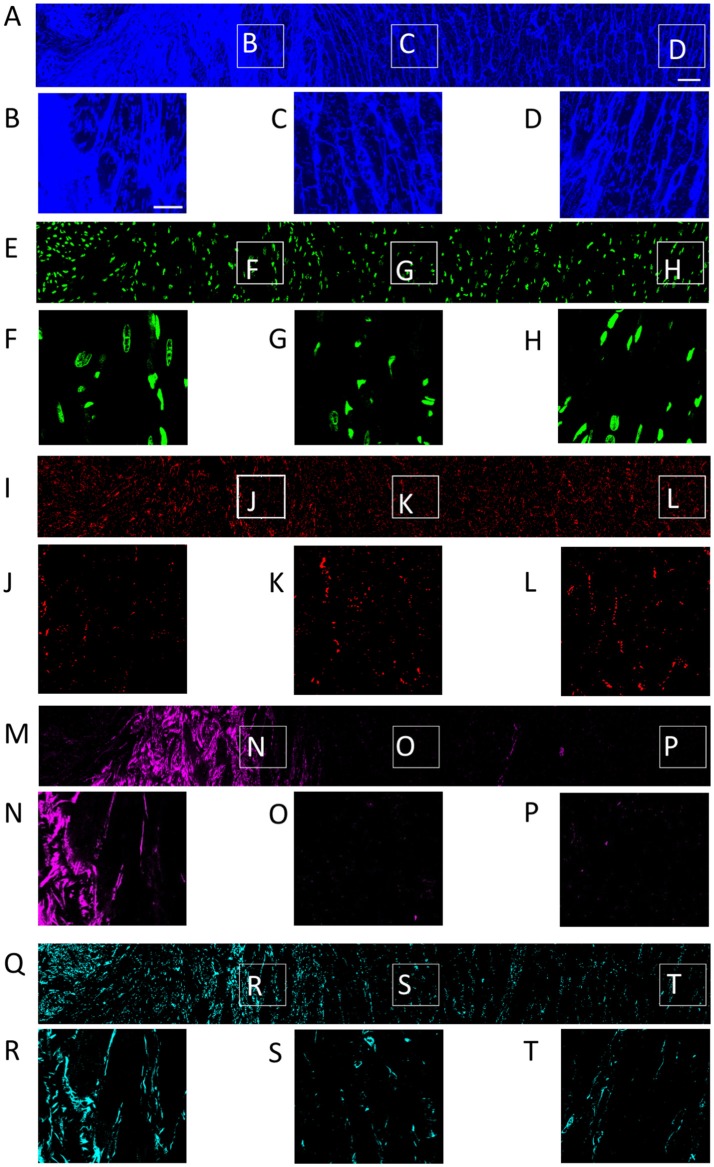
Distribution of **(A)** WGA, **(E)** DAPI, **(I)** Cx43, **(M)** α-SMA, and **(Q)** vimentin in a single slice of an image stack from MI heart. Scar to region farthest from scar is shown from left to right. Zoom-in of regions identified in panel **(A)** are shown for each channel. Scale bar in **(A)** 50 μm. Applies to **(E,I,M,Q)**. Scale bar in **(B)** 20 μm. Applies to **(C,D, F–H, J–L, N–P, R–T)**.

**Figure 2:**
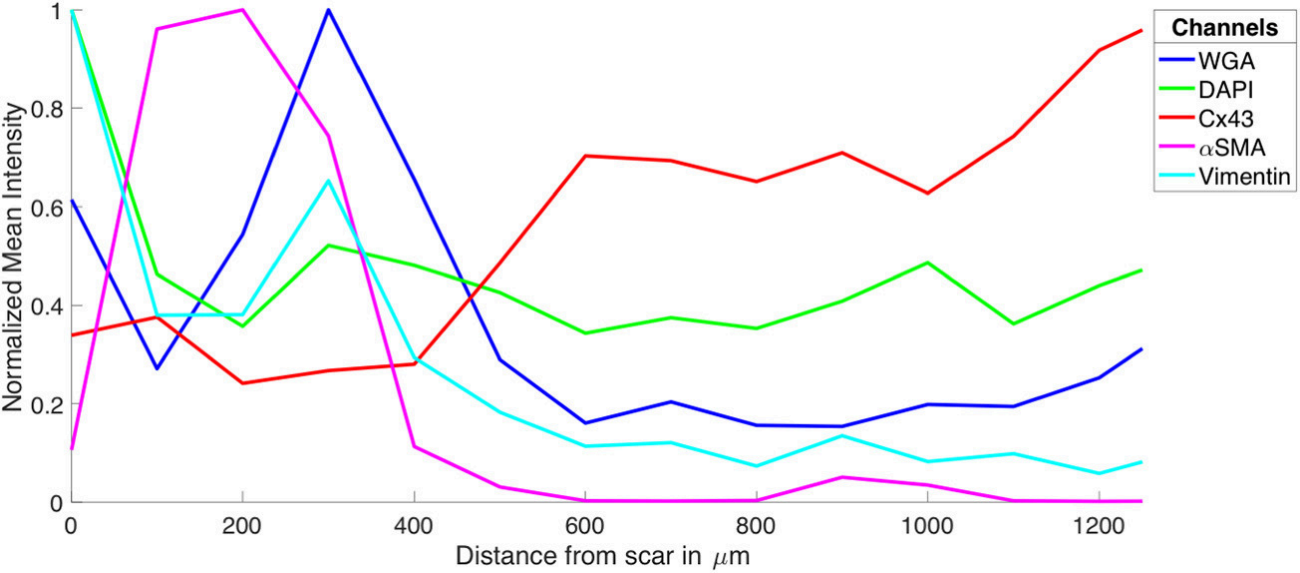
Intensity profiles of DAPI, Cx43, WGA, α-SMA, and vimentin through MI to MI border zone to distal tissue. The profiles were generated from the image stacks represented in Figure 1.

We studied tissue remodeling in the MI heart using the methods for fluorescent labeling and 3D tile scanning confocal microscopy as described above. Overview images with a field of view of 1495.8 × 204.8 × 60.6 μm and zooms into specific regions are shown in Figure 1. Furthermore, we calculated profiles of fluorescence intensities to describe the spatial relationship of tissue constituents vs. infarct distance in Figure 2.

The tile scanning covered part of the infarct scar and adjacent ventricular myocardium exhibiting different types and degrees of remodeling. Imaging of WGA-labeled tissue provided insights into the remodeling of extracellular matrix, which was extensively increased in the infarct scar and border zone (Figure 2). In comparison, myocardium at more distant sites exhibited reduced WGA signal, indicating normal amounts of extracellular space. Myocytes in close proximity to the MI (Figure 1B) were hypertrophic and, as a result of interstitial fibrosis, less densely packed vs. more distant myocytes (Figures 1C,D).

Images of DAPI yielded information on the distribution of cell nuclei (Figures 1E–H). A high nucleus density was revealed within the infarct scar (Figure 2), reflecting increased amounts of small cells, e.g. fibroblasts, myofibroblasts and macrophages. The intensity of Cx43 increased with infarct distance (Figures 1J–L). Accordingly, minima and maxima of Cx43 intensity were visible for scar and distant myocardium, respectively (Figure 2).

The distribution of α-SMA was heterogeneous with a significant increase near the infarct border zone (Figures 1M–P). Also, small regions of increased α-SMA intensity were found throughout myocardium distant from the scar. While α-SMA is a marker not only of myofibroblasts, but also of smooth muscle cells, for instance in the wall of blood vessels, the spatial distribution of α-SMA signal in the infarct border zone (Figure 1M) suggests abundance of myofibroblasts.

The spatial distribution of vimentin signal was qualitatively similar to the distribution of DAPI and α-SMA signal (Figures 1Q–T). However, the maximum of vimentin signal was found slightly more distant from the infarct border (250 μm) than the maximum of α-SMA signal (150 μm). In cardiac tissues, vimentin is a marker of fibroblasts, myofibroblasts, smooth muscle and endothelial cells.

For the subsequently described 3D reconstructions of tissue microstructure, we applied segmentations of capillaries to avoid that vessel associated vimentin-positive cells are misclassified as fibroblasts. We identified fibroblasts as non-vessel associated, vimentin-positive and α-SMA-negative. Based on these criteria, sparse vimentin signal in Figure 1T and Figure S1 points at the presence of fibroblasts.

### Three-dimensional reconstruction and analyses of myocardium in control and MI animals

**Figure 3:**
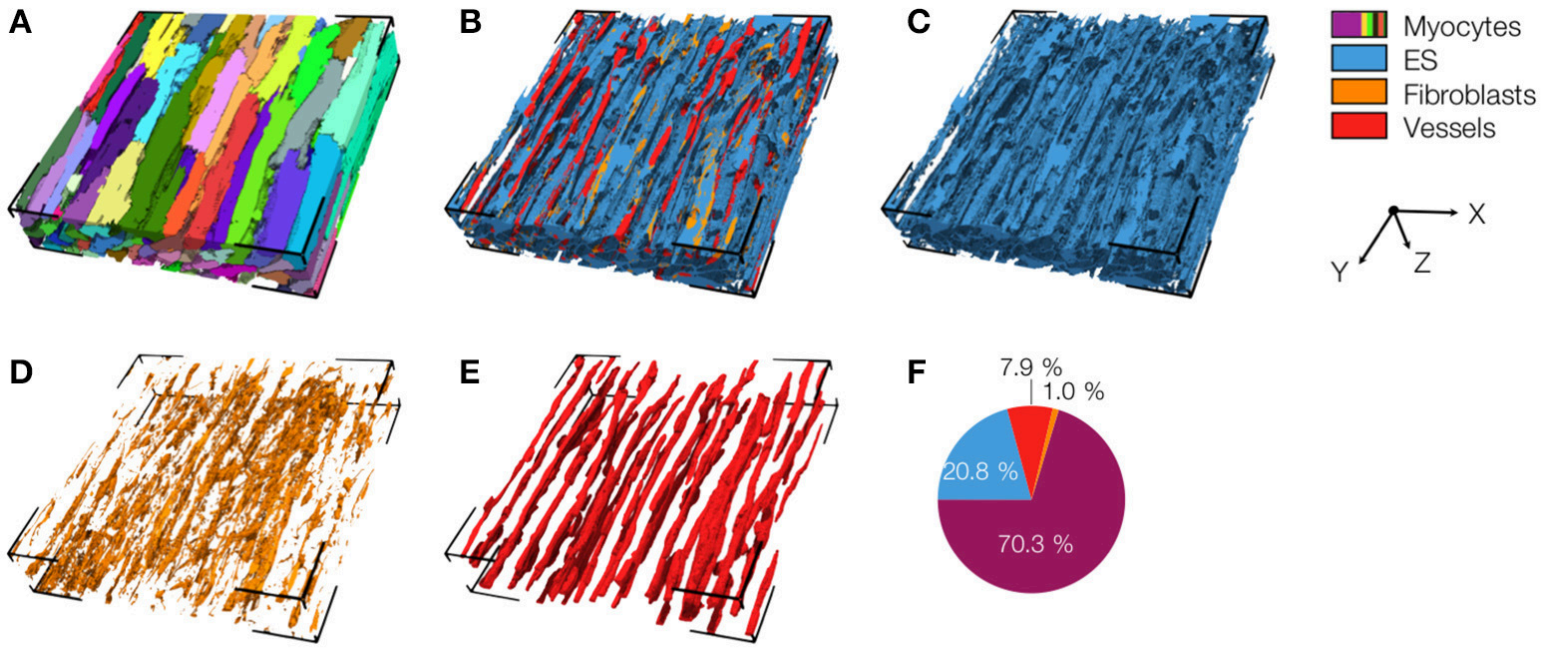
3D reconstruction of control myocardium with **(A)** cardiomyocytes, **(B)** complementary tissue constituents, **(C)** extracellular space, **(D)** fibroblasts, and **(E)** vessels. Volume fractions of tissue constituents are shown in **(F)**. The reconstruction has a size of 204.8 × 204.8 × 41.2 μm.

We acquired 3D image stacks from myocardium of the LV free wall in the normal heart (animals: 5, images: 8) and of region 1 (proximal, animals 4, images: 5), region 2 (adjacent, animals 6, images: 6), and region 3 (distal, animals: 4, images: 5) of the MI hearts. Raw sections from example image stacks are presented in the Figures S1–S4, respectively. Corresponding preprocessed images are shown in Figures S5–S8. An exemplary reconstruction from a control heart is shown in Figure 3. As in our prior work (Schwab et al., 2013; Seidel et al., 2013, 2016), the reconstruction recapitulates major features of ventricular myocardium, in particular, the dense arrangement and high volume fraction of myocytes (70.30%). While size and shape of myocytes was diverse, their long axes were approximately parallel. Myocytes were in close proximity to small blood vessels with a diameter and wall composition characteristic for capillaries. Capillaries were in general aligned with myocyte long axes. The reconstruction describes also the distribution of vimentin-positive cells, supposedly fibroblasts, which exhibit only a small volume fraction (1.03%). The tissue presented only marginal regions with α-SMA signal (0.02%), suggesting absence of myofibroblasts.

**Figure 4:**
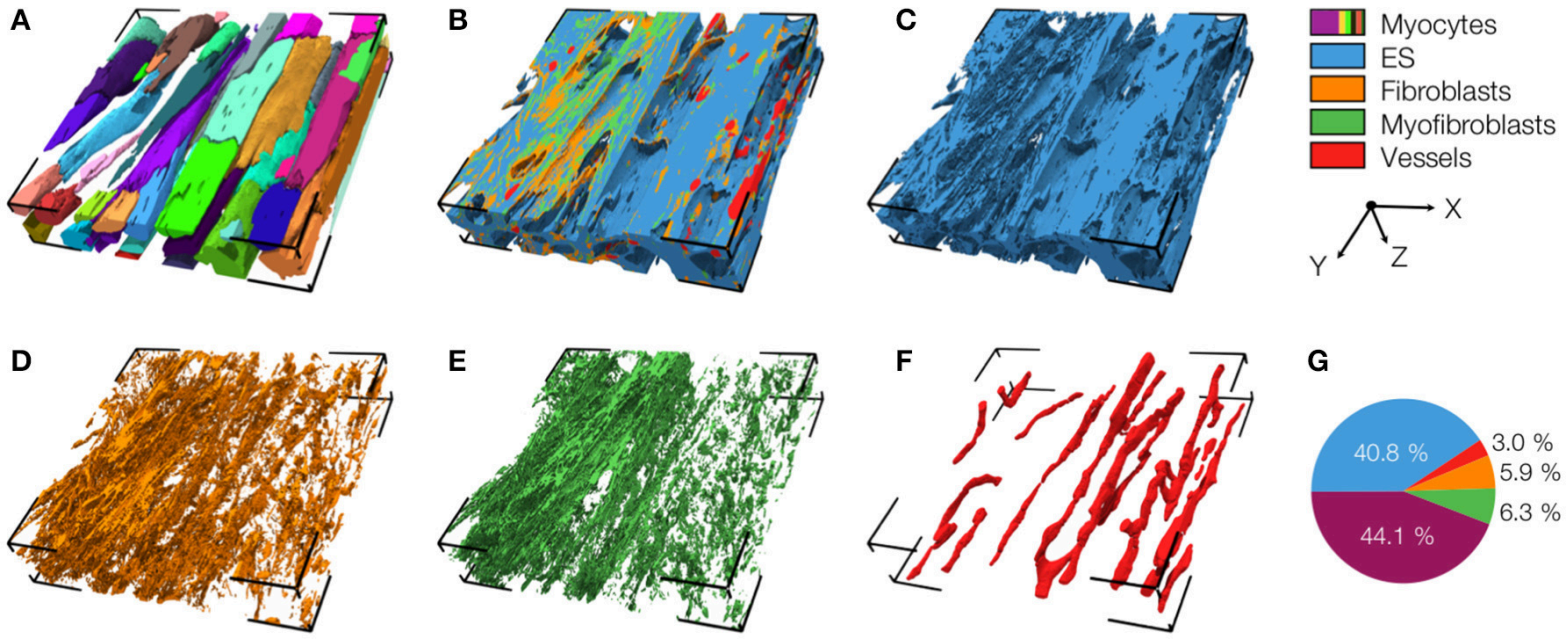
3D reconstruction of tissue in region 1 with **(A)** cardiomyocytes, **(B)** complementary tissue constituents, **(C)** extracellular space, **(D)** fibroblasts, **(E)** myofibroblasts, and **(F)** vessels. Volume fractions of tissue constituents are shown in **(G)**. The reconstruction has a size of 204.8 × 204.8 × 41.2 μm.

An exemplary reconstruction from the MI border zone (region 1) is shown in Figure 4. The reconstruction highlights various types of MI-associated tissue remodeling including reduced density and hypertrophy of myocytes, decreased capillary density, increased volume fractions of extracellular space and fibroblasts as well as the abundance of myofibroblasts.

**Figure 5:**
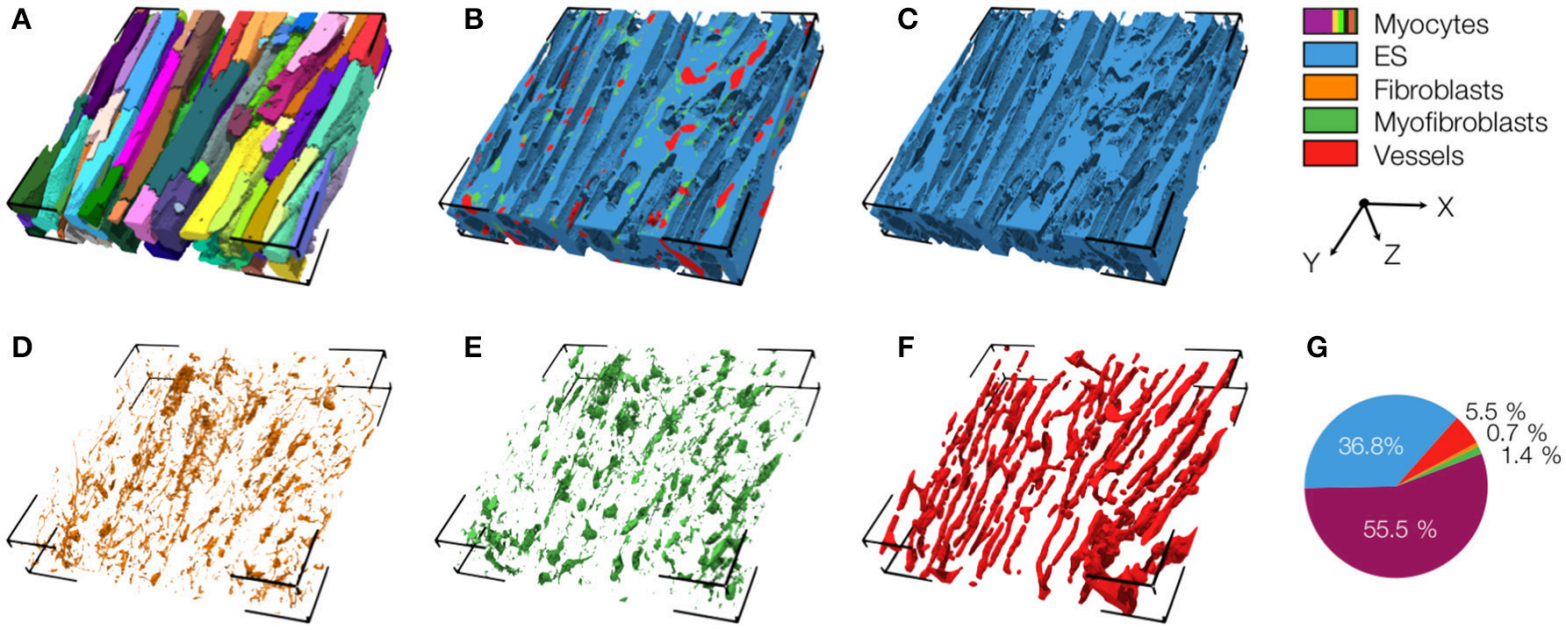
3D reconstruction of tissue in region 3 with **(A)** cardiomyocytes, **(B)** complementary tissue constituents, **(C)** extracellular space, **(D)** fibroblasts, **(E)** myofibroblasts, and **(F)** vessels. Volume fractions of tissue constituents are shown in **(G)**. The reconstruction has a size of 204.8 × 204.8 × 41.2 μm.

We also present a reconstruction of distal tissue (region 3, ~1 mm infarct distance) in the MI heart (Figure 5). In many aspects, the tissue microstructure appeared similar as for control tissue. *V*_*myo*_ and *V*_*e*_ were only marginally different. In contrast to control tissue, the region exhibited α-SMA positive cells, suggesting the presence of myofibroblasts.

**Figure 6:**
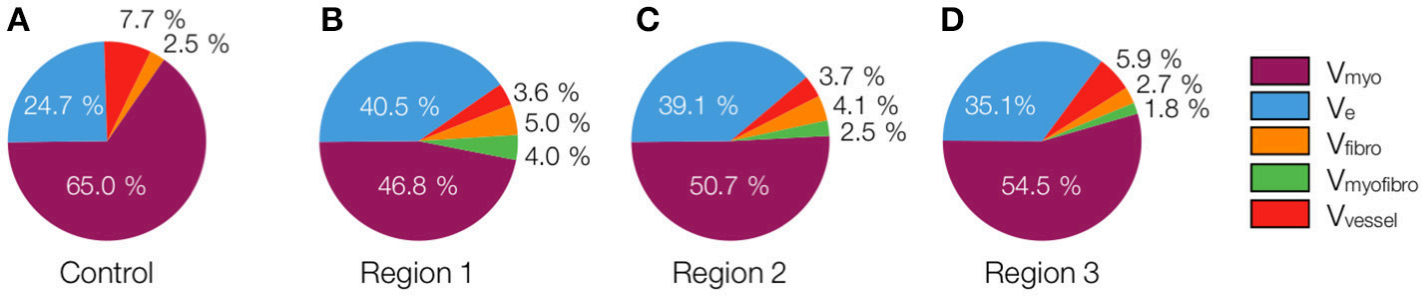
Pie charts of volume fractions in **(A)** control, **(B)** region 1, **(C)** region 2, and **(D)** region 3. For statistical information and significance of differences see Table 1.

The 3D reconstructions allowed us to calculate *V*_*myo*_, *V*_*fibro*_, *V*_*myofibro*_, *V*_*vessel*_, and *V*_*e*_. From 3D reconstructions of interlaminar clefts (see examples in Figure S9), we estimated *V*_*cleft*_. The volume fractions are summarized in Figure 6 and Table 1. The analyses revealed an increase of *V*_*e*_, accompanied by decreased *V*_*myo*_ in regions 1 and 2 vs. control tissue. We also noted an increase of *V*_*myofibro*_ in regions 1–3 vs. control. *V*_*vessel*_ in regions 1 and 2 was decreased vs. control. In our experimental groups, *V*_*fibro*_ exhibited a high standard deviation vs. mean.

**Table 1:** Calculated volume fractions of tissue constituents of control and MI hearts.

	***V_*myo*_* [%]**	***V_*e*_* [%]**	***V_*cleft*_* [%]**	***V_*vessels*_* [%]**	***V_*fibro*_* [%]**	***V_*myofibro*_* [%]**
Control (*n* = 8)	65.03 ± 3.6	24.68 ± 3.05	3.95 ± 4.84	7.71 ± 2.15	2.48 ± 1.11	0.09 ± 0.08
Region 1 (*n* = 5)	46.80 ± 4.49^*^	40.53 ± 6.33^*^	5.14 ± 3.78	3.64 ± 1.95^*^	5.00 ± 3.25	4.03 ± 3.46^*^
Region 2 (*n* = 6)	50.68 ± 10.87^*^	39.06 ± 10.44^*^	5.56 ± 5.40	3.65 ± 1.39^*^	4.12 ± 3.75	2.48 ± 1.95^*^
Region 3 (*n* = 5)	54.51 ± 10.95	35.12 ± 10.16	12.84 ± 8.29	5.87 ± 1.34	2.69 ± 2.36	1.81 ± 0.67^*^

Values are presented as mean ± SD. Number of image stacks: n. Statistical significance vs. control are marked with ^*^.

**Figure 7:**
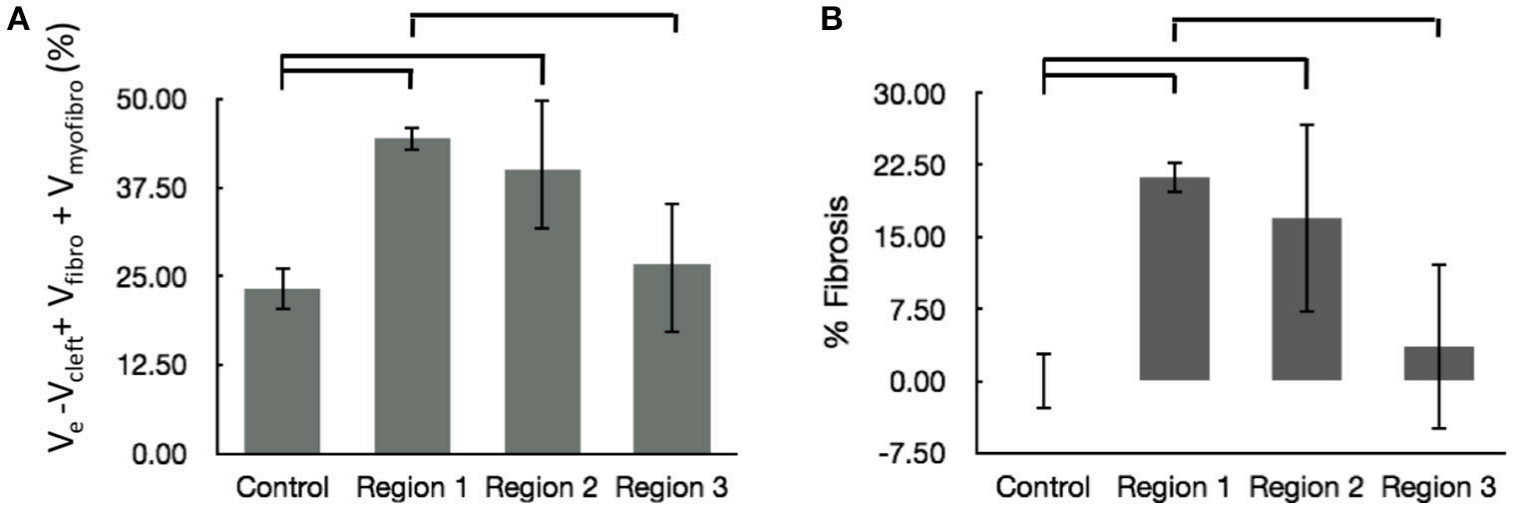
Evaluation of fibrosis. **(A)** Sum of fractional volumes of domains contributing to fibrosis. **(B)** Degree of fibrosis in proximal, adjacent and distal regions to the MI calculated based on Equation (1). Brackets mark significant differences between groups.

We calculated the volume fractions of fibrotic tissue for control and MI regions 1–3 (Figure 7A). Regions 1 and 2 of MI hearts exhibited increased fraction of fibrotic tissue when compared with control. Additionally, the fibrotic tissue in region 3 was significantly lower than that in Region 1. The regional degree of fibrosis calculated from Equation 1 is presented in Figure 7B. The degree of fibrosis was significantly higher in region 1 and 2, but not in region 3, with respect to control. The degree ranged from 21.21 ± 1.73% in region 1 to 3.58 ± 8.64% in region 3. While fibrotic tissue was abundant close to the infarct, we observed high variabilities in more distant regions 2 and 3, as seen from high SD in those regions (Figure 7B).

### Image-based calculation of extracellular conductivity of control and MI myocardium

We applied 3D reconstructions of tissue microstructure for computational measurements of extracellular conductivities. In these reconstructions, the orientation of myocytes was approximately parallel to the y-axis.

**Figure 8:**
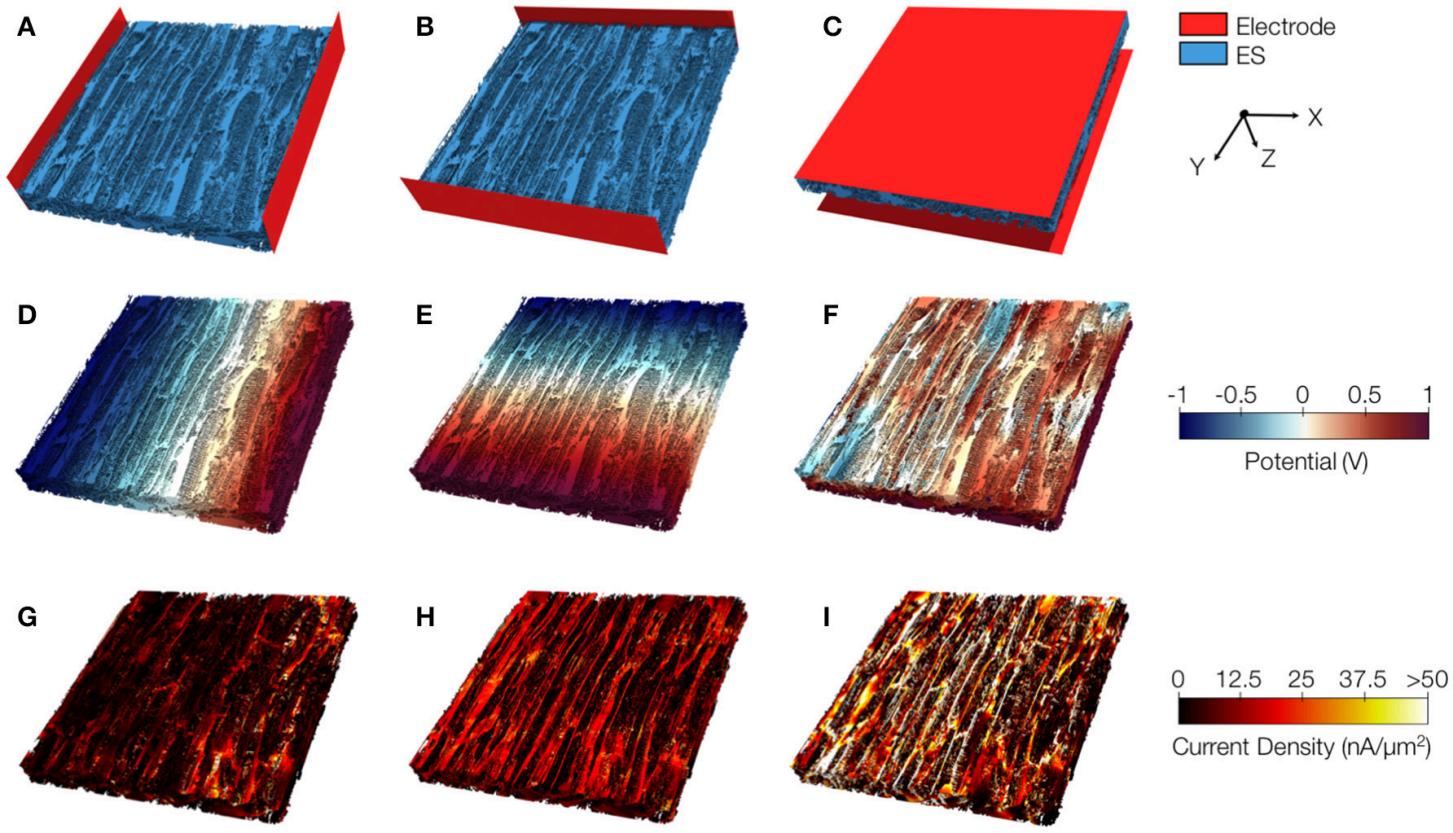
Image-based computation of extracellular conductivity tensor for control tissue. 3D-reconstructions of the extracellular space with modeled electrodes for applying voltages in **(A)** x, **(B)** y, and **(C)** z-direction. Only half of the image stacks is shown. **(D–F)** Calculated potential distribution corresponding to **(A–C)**. **(G–I)** Calculated current density corresponding to **(D–F)**.

Exemplary conductivity models of control tissue with assigned electrodes for creating potential gradients in x-, y-, and z-direction are shown in Figures 8A–C, respectively. The electrodes were applied to assign Dirichlet boundary conditions. Figures 8D–F present the calculated electrical potential distributions resulting from setting the Dirichlet boundary conditions to ±1 *V*. The calculation of the electrical field is limited to the extracellular space. Figures 8G–I depict current densities calculated from the electrical potential distributions for the exemplary control. Calculated conductivities were σ_*e,l*_ = 0.34 S/m, σ_*e,t*_ = 0.10 S/m, and σ_*e,n*_ = 0.10 S/m for this tissue reconstruction.

**Figure 9:**
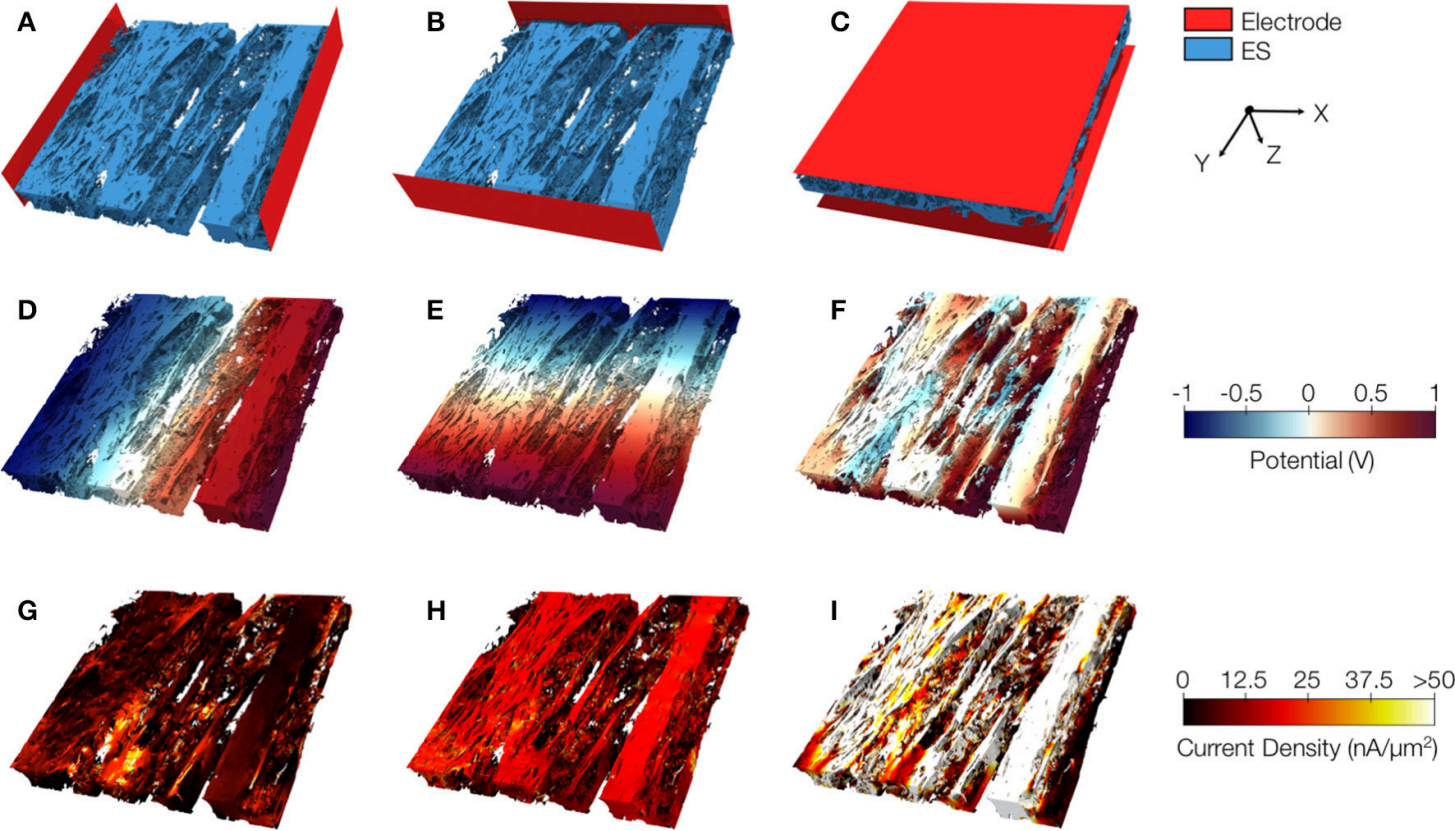
Image-based computation of extracellular conductivity tensor for tissue in region 1. 3D-reconstructions of the extracellular space with modeled electrodes for applying voltages in **(A)** x, **(B)** y, and **(C)** z-direction. Only half of the image stacks is shown. **(D–F)** Calculated potential distribution corresponding to **(A–C)**. **(G–I)** Calculated current density corresponding to **(D–F)**.

**Figure 10:**
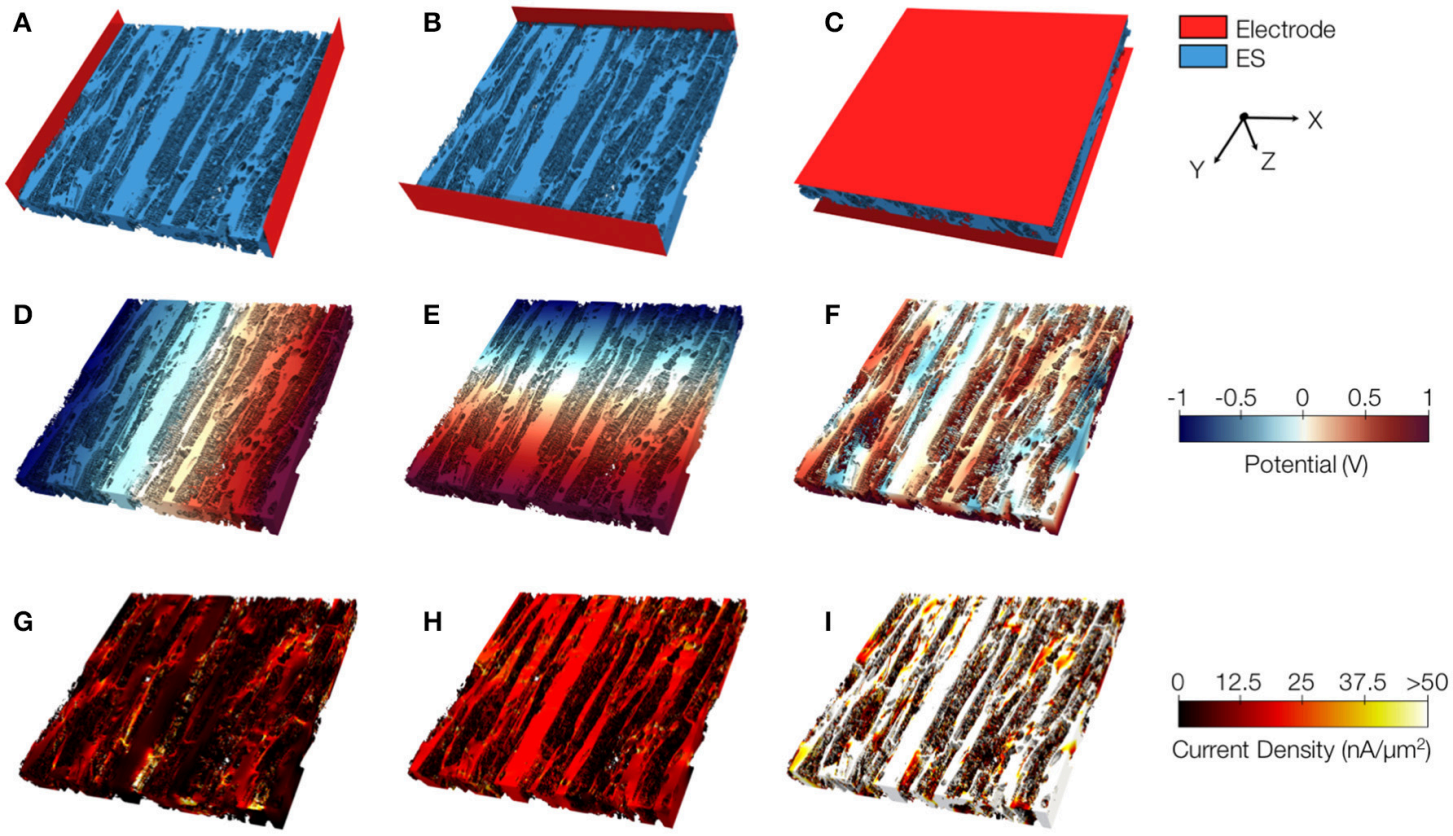
Image-based computation of extracellular conductivity tensor for tissue in region 3. 3D-reconstructions of the extracellular space with modeled electrodes for applying voltages in **(A)** x, **(B)** y, and **(C)** z-direction. Only half of the image stacks is shown. **(D–F)** Calculated potential distribution corresponding to **(A–C)**. **(G–I)** Calculated current density corresponding to **(D–F)**.

We present similar illustrations for tissue reconstructions from region 1 and 3 in Figures 9, and 10, respectively. Calculated conductivities for the tissue reconstruction for region 1 (σ_*e,l*_ = 0.69 S/m, σ_*e,t*_ = 0.51 S/m, and σ_*e,n*_ = 0.20 S/m) were larger than for control. Similarly, conductivities for the tissue reconstruction for region 3 were larger (σ_*e,l*_ = 0.53 S/m, σ_*e,t*_ = 0.41 S/m, and σ_*e,n*_ = 0.15 S/m) vs. control. The tissue reconstructions include pronounced interlaminar clefts associated with high current densities for application of voltage gradients in z-direction (Figures 9I and 10I).

**Figure 11:**
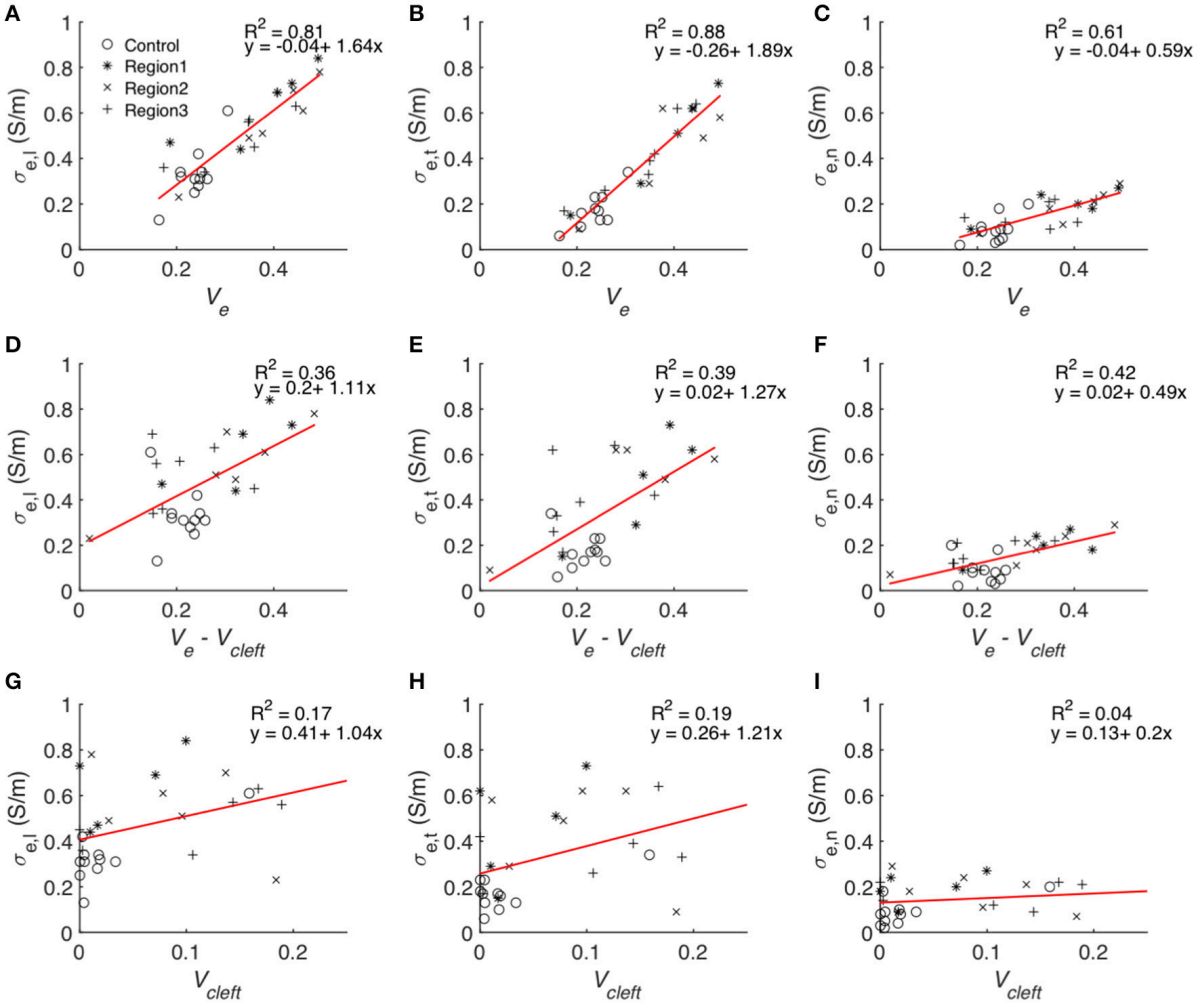
Statistical analyses of the relationship between volume fractions of tissue constituents and calculated conductivities. Each symbol represents an image-based computation of conductivity. Linear regression modeled the relationship between *V*_*e*_ and **(A)** σ_*e,l*_, **(B)** σ_*e,t*_ and **(C)** σ_*e,n*_, *V*_*e*_–*V*_*cleft*_ and **(D)** σ_*e,l*_, **(E)** σ_*e,t*_ and **(F)** σ_*e,n*_ as well as between *V*_*cleft*_ and **(G)** σ_*e,l*_, **(H)** σ_*e,t*_, and **(I)** σ_*e,n*_.

Statistical analysis of the computational measurements revealed an approximately linear relationship between *V*_*e*_ and the extracellular conductivities (Figure 11). The goodness of the fit to a linear model (assessed by *R*^2^) as well as slopes of the regression line of *V*_*e*_–σ_*e,l*_ and *V*_*e*_–σ_*e,t*_ were higher than for *V*_*e*_–σ_*e,n*_. Regression analysis of *V*_*cleft*_ as well as *V*_*e*_–*V*_*cleft*_ and extracellular conductivities revealed a weak fit (*R*^2^ < 0.5) to a linear model. Slopes of the regression line of *V*_*cleft*_–σ_*e,l*_ and *V*_*cleft*_–σ_*e,t*_ were higher than for *V*_*e*_–σ_*e,l*_ and *V*_*e*_–σ_*e,t*_. However, the slope for *V*_*e*_–σ_*e,n*_ was similar as for *V*_*cleft*_–σ_*e,n*_.

The goodness of fit for linear regression between *V*_*cleft*_ and *V*_*e*_–*V*_*cleft*_ was only 0.018. Multiple linear regression analysis with the two predicting variables *V*_*e*_–*V*_*cleft*_ and *V*_*cleft*_ resulted in almost identical *R*^2^ (Table S2) as presented in Figures 11A–C.

We present statistical data on measured extracellular conductivities for control and MI tissues in Table 2. Conductivities in fiber, transverse and sheet direction in region 1 were larger than corresponding conductivities in the control heart. Noteworthy is the pronounced increase of σ_*e,t*_ in all MI regions vs. control (~2.4- to 2.7-fold). Conductivity in transverse direction of all the three MI regions and in fiber direction of region 1 were significantly increased vs. corresponding conductivities in control. The changes of conductivities were reflected in the decreased ratios σ_*e,l*_/σ_*e,t*_ and increased ratios σ_*e,t*_/σ_*e,n*_ in MI vs. control tissue.

**Table 2:** Calculated extracellular conductivities and their ratios for control and MI tissues.

	**σ_*e,l*_*[S/m]***	**σ_*e,t*_*[S/m]***	**σ_*e,n*_*[S/m]***	**σ_*e,l*_/σ_*e,t*_**	**σ_*e,t*_/σ_*e,n*_**
Control (*n* = 8)	0.36 ± 0.11	0.17 ± 0.07	0.10 ± 0.06	2.07	1.68
Region 1 (*n* = 5)	0.63 ± 0.17^*^	0.46 ± 0.23^*^	0.20 ± 0.07	1.38	2.35
Region 2 (*n* = 6)	0.55 ± 0.19	0.45 ± 0.22^*^	0.18 ± 0.08	1.23	2.45
Region 3 (*n* = 5)	0.51 ± 0.11	0.41 ± 0.17^*^	0.16 ± 0.06	1.25	2.48

Conductivities are presented as mean value ± SD. Number of 3D reconstructions: n. Statistical significance vs. control are marked with ^*^.

## Discussion

Parameters of mathematical models crucially determine outcomes of computational simulations. It is thus surprising that for simulations of cardiac conduction our knowledge on modeling parameters and their variability is very limited, in particular for diseased tissues. Here, we addressed this problem and presented steps toward imaging-based measurement of parameters for models of electrical conduction in the normal and diseased heart.

Large overview images covering the scar and regions of interest illustrated pronounced changes in the distribution and volume fractions of different tissue constituents in the MI heart. We focused on quantifying volume fractions of tissue constituents and calculating extracellular conductivities. Fluorescent labeling and confocal microscopy allowed us to generate 3D reconstructions of tissue microstructure in control and diseased hearts. We analyzed these reconstructions to provide quantitative information on remodeling of volume fractions of tissue constituents in MI. Our approach quantified decreased *V*_*myo*_ accompanied by increased *V*_*e*_ proximal and adjacent to the scar (regions 1 and 2) vs. control tissue. We propose that the measured volume fractions provide a solid foundation for parameterization of models of cardiac conduction. For instance, the measurements can be used to adjust volume fractions of the intracellular and extracellular domains in multidomain modeling (Sachse et al., 2009). Similarly, the provided information on *V*_*fibro*_ and *V*_*myofibro*_ in control and MI tissue can be used to inform the design of computational studies of cardiac conduction. For example, the study design can reflect the presence of myofibroblasts found in MI regions, which may alter myocyte electrophysiology and impulse propagation as a result of myocyte-myofibroblast interactions. Moreover, this information can help to constrain the ranges of parameter sensitivity studies on effects of fibroblasts and myofibroblasts.

We applied the tissue reconstructions to calculate extracellular conductivity tensors in control and MI myocardium at the submillimeter scale (Table 2). Measurements of cardiac tissue conductivities using conventional electrode-based systems have not been performed at this scale. While single-cell membrane electrophysiology is accessible by patch clamp experiments, and has therefore been studied extensively, it remains difficult to measure conductivity parameters of myocardial tissue, especially on a small scale. Thus, mathematical models have been used to estimate these parameters. A comparison of our computational results with published measurements and model-based estimations of extracellular conductivities is presented in Table 3. Noteworthy, most prior measurements were obtained from normal tissues. Only a small number of studies presented all three components of the extracellular conductivity tensor. The spread of published values is large, which arguably reflects differences in experimental approach, species and tissue regions. Our result of σ_*e,l*_ = 0.36 ± 0.11 S/m in LV tissue of control rabbit is close to a prior measurement of 0.40 S/m from RV papillary muscle in the same species (Kleber and Riegger, 1987).

**Table 3:** Extracellular conductivities for control and MI tissue.

**References**	**Tissue type/location**	**Origin**	**σ_e,l_ [S/m]**	**σ_e,t_ [S/m]**	**σ_e,n_ [S/m]**
This study	Rabbit LV	Imaging	0.36	0.17	0.10
Clerc, 1976	Calf RV trabecula	Measurement	0.63	0.24	
Hand et al., 2009		Model	0.30	0.16	
Hooks et al., 2002	Rat LV	Model	0.26	0.11	0.11
Kleber and Riegger, 1987	Rabbit RV papillary	Measurement	0.40		
Roberts et al., 1979	Canine LV	Measurement	0.22	0.13	
Roberts and Scher, 1982	Canine LV	Measurement	0.12	0.08	
Schwab et al., 2013^+^	Rabbit LV	Imaging	0.26	0.22	0.13
Stinstra et al., 2005		Model	0.21	0.06	
**MI**					
	Rabbit LV				
This Study	0–0.2 mm 0.25–0.75 mm 1–1.25 mm	Imaging	0.63 0.55 0.51	0.46 0.45 0.41	0.20 0.18 0.16
	Rabbit LV				
Schwab et al., 2013^+^	0.5–5.5 mm 7.5–12.5 mm 12.5–17.5 mm 17.5–22.5 mm	Imaging	0.26 0.40 0.28 0.28	0.20 0.29 0.27 0.19	0.17 0.31 0.17 0.17

+ *Myocyte sheets and interlaminar clefts were not identified. Thus, conductivities are ordered by magnitude*.

An interesting finding was that the spread of calculated conductivities (see SD values in Table 2) is large, suggesting pronounced spatial heterogeneity of conductivity at the submillimeter scale for all our experimental groups. In general, computational models of cardiac conduction do not account for these spatial heterogeneities and apply homogeneous conductivity values. The new data presented in Table 2 establish a basis for computational studies accounting for heterogeneous distribution of conductivities.

The contribution of heterogeneities in extracellular conductivities and tissue composition in various cardiac diseases may be assessed computationally using the approach described in this study. While our study focused on applications related to simulations of cardiac conduction, further potential applications include parameter estimation for simulations of cardiac biomechanics. It is well established that tissue remodeling such as fibrosis affects active and passive mechanical tissue properties. We suggest that the 3D reconstructions provide a basis for simulation of cardiac biomechanics at the submillimeter scale.

An important finding of our study is that *V*_*e*_ showed high variability at submillimeter scale. This variability in part results from the distribution of interlaminar clefts: They appear between lamina consisting of 3–5 myocyte layers. Assuming a myocyte height of 14 μm (Lasher et al., 2009) and an interstitial thickness of 3 μm, one would expect one cleft every 48–82 μm. Stack width in our images was ~200 μm. Thus, depending on the imaged region and lamina width, two to four clefts were present in one stack, increasing variability in cleft volume fraction.

Differences of volume fractions found between region 3 and control were not significant except for myofibroblasts, suggesting that at distances larger than 1 mm from the infarct, tissue composition tends to be largely unaffected by the infarct. Previous studies using rabbit MI models, but different methods of analysis (Driesen et al., 2007; Seidel et al., 2017), have shown a similar intactness of tissue distal from the scar. Interestingly, at 2 weeks after MI in rat, the transition from the infarcted to normal tissue occurred within only 200–300 μm (Rutherford et al., 2012), whereas at 2, 5, and 8 weeks of MI in sheep, fibrotic alterations were found also in myocardium remote from the infarct (Jackson et al., 2002). Thus, the width of the peri-infarct zone and remodeling seems species-dependent. We suggest that our methodology will be useful to characterize species-specific remodeling.

We note that many approaches have been developed to quantify fibrosis in histological sections. A common measure is quantification of collagen after Masson's trichrome staining in thin tissue sections. Also, WGA, which stains the extracellular matrix and the pericellular matrix, i.e., the glycocalyx, has been proposed for identification of collagen (Soderstrom, 1987; Emde et al., 2014). However, fibrosis is defined as the formation of excess connective tissue, which includes not only collagen and other proteins of the extracellular matrix, but also cells of fibrous connective tissue, such as fibroblasts and myofibroblasts. To account for this definition and to quantify fibrosis more comprehensively, the presented study introduces a quantitative measure of cardiac fibrosis (Equation 1). We calculated the degree of fibrosis as the increased volume fraction of fibrotic tissue constituents in a region of interest vs. control. This included the increase of volume fractions of extracellular space without clefts, fibroblasts and myofibroblasts. As an alternative for comparing the degree of fibrosis between different tissue types, Equation (1) can be normalized by the volume fraction of non-fibrotic tissue constituents in control. The major contributor to fibrosis was increased *V*_*e*_. In our calculation, we excluded volumes of interlaminar clefts, because those exhibited low WGA values indicating only marginal presence of collagen. We did not find increases of WGA intensity in clefts in MI. Our measure suggests that fibrosis is a decreasing function of distance from the infarct scar.

The presented conductivity analyses are based on approaches that we introduced earlier (Schwab et al., 2013). We note several important differences: The investigated tissue samples were more proximal to the MI than in our previous study (Table 3), which allowed us to describe more pronounced remodeling. Compared to the previously used threshold-based approach to segment image stacks into 4 domains (Table 3), we created more refined and accurate reconstructions of the tissue composition. This allowed us to augment our conductivity model with information about six domains. In particular the information on interlaminar clefts allowed us novel analyses. These clefts were not visible in many previous studies, because conventional mounting methods cause compression of tissue preparations. In the presented computational simulations, we increased the spatial resolution from 800 nm to 200 nm. Furthermore, an increased number of studied animals allowed us to perform statistical tests, which was not possible in previous work.

Several assumptions underlie the presented conductivity measurements. The exclusion of fibroblast and myofibroblast domains is motivated by the cell membrane of those cells, which poses a barrier for currents in the extracellular space. We excluded the vessel domain from the conductivity model with the reasoning that endothelial cells in the capillary wall represent a high resistivity for extracellular currents (Olesen and Crone, 1983; Stinstra et al., 2005). The myocyte segmentation was masked with a thresholded WGA image to conservatively reproduce the surface region between the extracellular space and myocytes. This step also added the transverse tubular system of the myocytes to the conductivity model. However, due to the simple structure of the transverse tubular system in rabbit (Savio-Galimberti et al., 2008), it is reasonable to assume that they do not contribute to extracellular conductivity in tissue.

Significant differences for several components of conductivity tensors were found in the border zone regions vs. control tissue. Longitudinal (σ_*e,l*_) and transverse (σ_*e,t*_) conductivities increased, whereas normal conductivities (σ_*e,n*_) did not change significantly. Because the increase in transverse conductivity was more pronounced, anisotropy (σ_*e,l*_/σ_*e,t*_) was reduced by nearly 50% in the border zone vs. control. These findings are important for simulations of MI-associated arrhythmia. It has been shown in several studies that reduced anisotropy can lead to conduction block and trigger arrhythmia, especially at the transition from high to low intracellular conductivity (Rohr et al., 1997; Seidel et al., 2010). We suggest that reduced extracellular anisotropy in the border zone contributes to these effects. Our study provides parameter ranges to test this hypothesis in computational models.

A major finding of this study was the approximately linear relationship between all components of the conductivity tensor and *V*_*e*_, which comprises *V*_*cleft*_. Indeed, linear regression analysis showed that *V*_*e*_ is an excellent predictor of the measured conductivities (Figure 11). In comparison, *V*_*cleft*_ alone and *V*_*e*_ without *V*_*cleft*_ were weak predictors. We explain these findings by an additive effect of *V*_*cleft*_ to the prediction of extracellular conductivity using *V*_*e*_ without *V*_*cleft*_ only. We also found that *V*_*cleft*_ and *V*_*e*_–*V*_*cleft*_ do not depend on each other. Excluding *V*_*cleft*_ from the predicting variable (Figures 11G–I) therefore leads to poor prediction of extracellular conductivity, despite the small volume contribution of the interlaminar clefts (Figure S9). These findings underpin the importance of structure-preserving mounting methods for image-based estimation of modeling parameters. We note that in addition to *V*_*e*_ knowledge on the orientation of myocyte fibers and lamina is necessary for predicting extracellular conductivities. Nevertheless, we suggest that the presented approach will enable efficient and accurate estimation of extracellular conductivity tensors from microscopic images.

## Limitations

We acknowledge limitations regarding tissue processing and imaging. Most of these limitations were discussed in our prior work (Lackey et al., 2011; Schwab et al., 2013; Seidel et al., 2013, 2016, 2017). Tissue remodeling after MI is a dynamic process, which is not completed after 3 weeks as well as species and size dependent (Pfeffer and Braunwald, 1990). Thus, volume fractions presented for regions 1 to 3 will differ at later or earlier stages of the remodeling and for other species. We note that vimentin and α-SMA are not specific for fibroblasts and myofibroblasts. Hence, the fibroblast and myofibroblast domains presented here include other non-myocytes. Fixation and processing of tissue biopsies may cause artifacts, for example tissue shrinking or tearing. It is unclear if these changes affect all tissue components to the same degree. Since tissue stability is especially low in interlaminar clefts, due to reduced collagen content, clefts may be deformed. However, in our earlier work we showed that in intact, living tissue, clefts appear very similar to those observed in fixed, WGA-labeled tissue slices and that compression-free mounting preserves tissue structure vs. conventional methods (Seidel et al., 2016). Similarly, the volume fraction of blood vessels may depend on perfusion pressure during fixation. This would constitute a systematic error because all hearts were perfused with the same system and pressure. The point spread function of a confocal microscope is anisotropic, thus blurring of, for example, the extracellular signal is more pronounced in laser light direction than transverse directions. With our imaging protocol, this might lead to higher conductivities in longitudinal and transverse vs. normal direction. Also, *V*_*e*_ might be overestimated near very thin intercellular spaces below the resolution limit. We reduced these issues by image deconvolution.

## Author contributions

FS and TS: designed the study; JG, AS, and FS: drafted the manuscript; TS: performed surgeries; AS and TS: acquired image data; JG and AS: analyzed the data. All authors developed software for this project, interpreted the data, critically revised the manuscript, and approved the version to be published.

### Conflict of interest statement

The authors declare that the research was conducted in the absence of any commercial or financial relationships that could be construed as a potential conflict of interest.
