# Supplementary material for: Confocal Microscopy-Based Estimation of Parameters for Computational Modeling of Electrical Conduction in the Normal and Infarcted Heart
Source: Front Physiol. 2018 Apr 4;9:239. doi: 10.3389/fphys.2018.00239 (PMC5893725; doi:10.3389/fphys.2018.00239)
Supplement: Supplementary file 1 [file Presentation1.PDF]

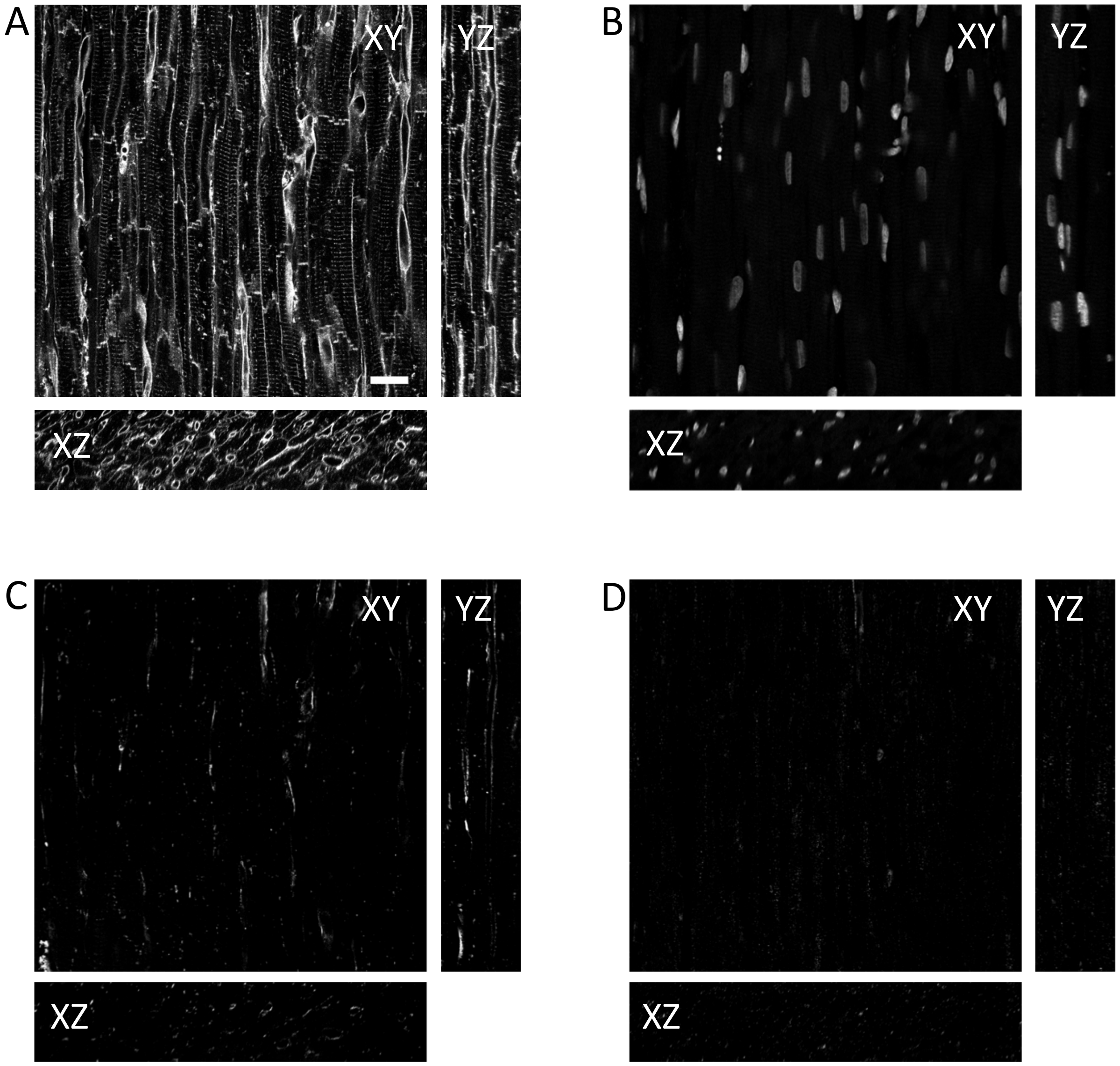

Figure S1: Raw images of central cross-sections from a 3D stack generated from control tissue labeled with **(A)** WGA, **(B)** DAPI, **(C)** vimentin, and **(D)** α-SMA**.** Scale bar in **(A)** has a length 20 µm and applies to **(B-D)**.

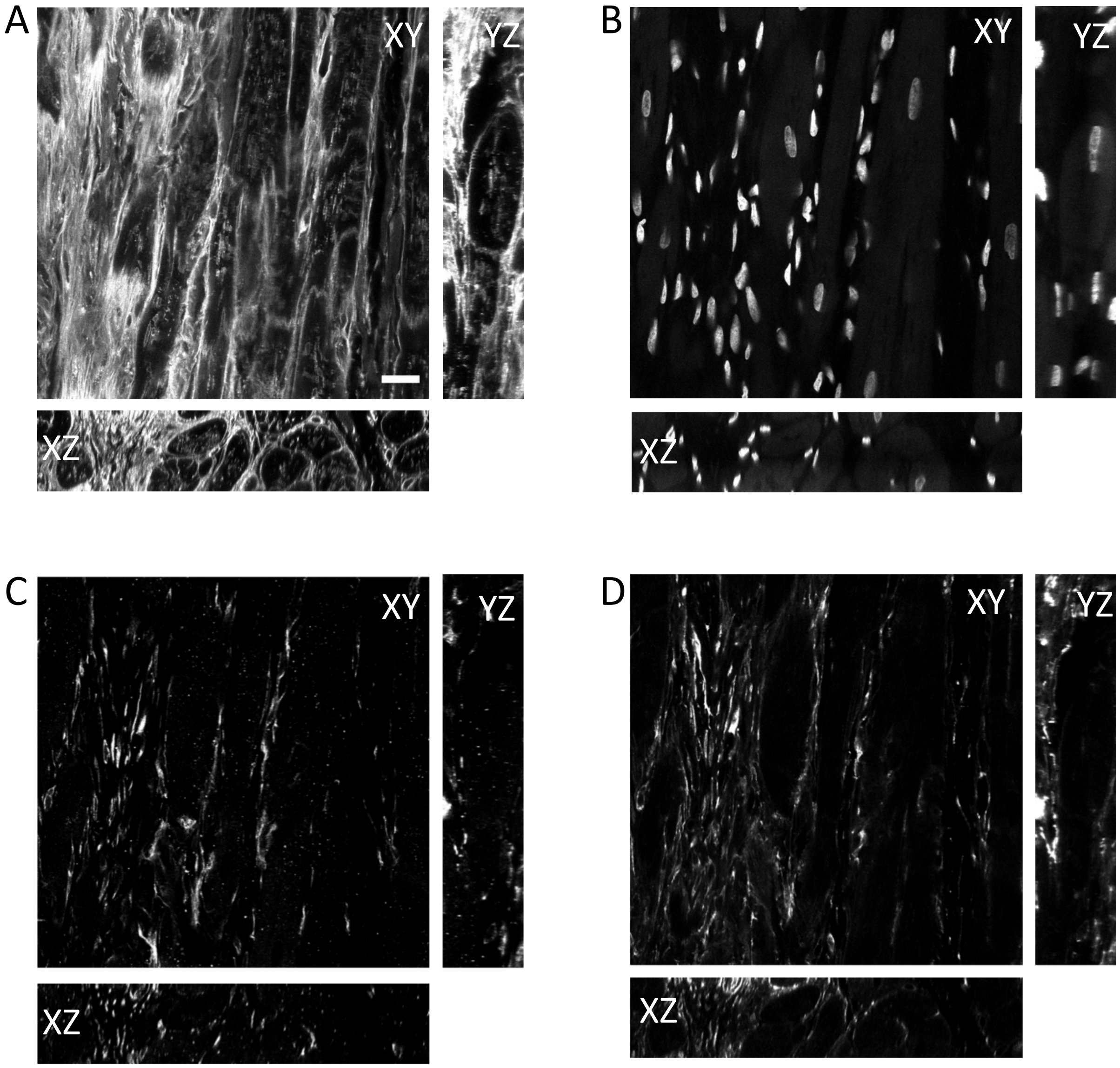

Figure S2: Raw images of central cross-sections from a 3D stack generated from region 1 of infarct tissue labeled with **(A)** WGA, **(B)** DAPI, **(C)** vimentin, and **(D)** α-SMA. Scale bar in **(A)** has a length 20 μm and applies to **(B-D)**.

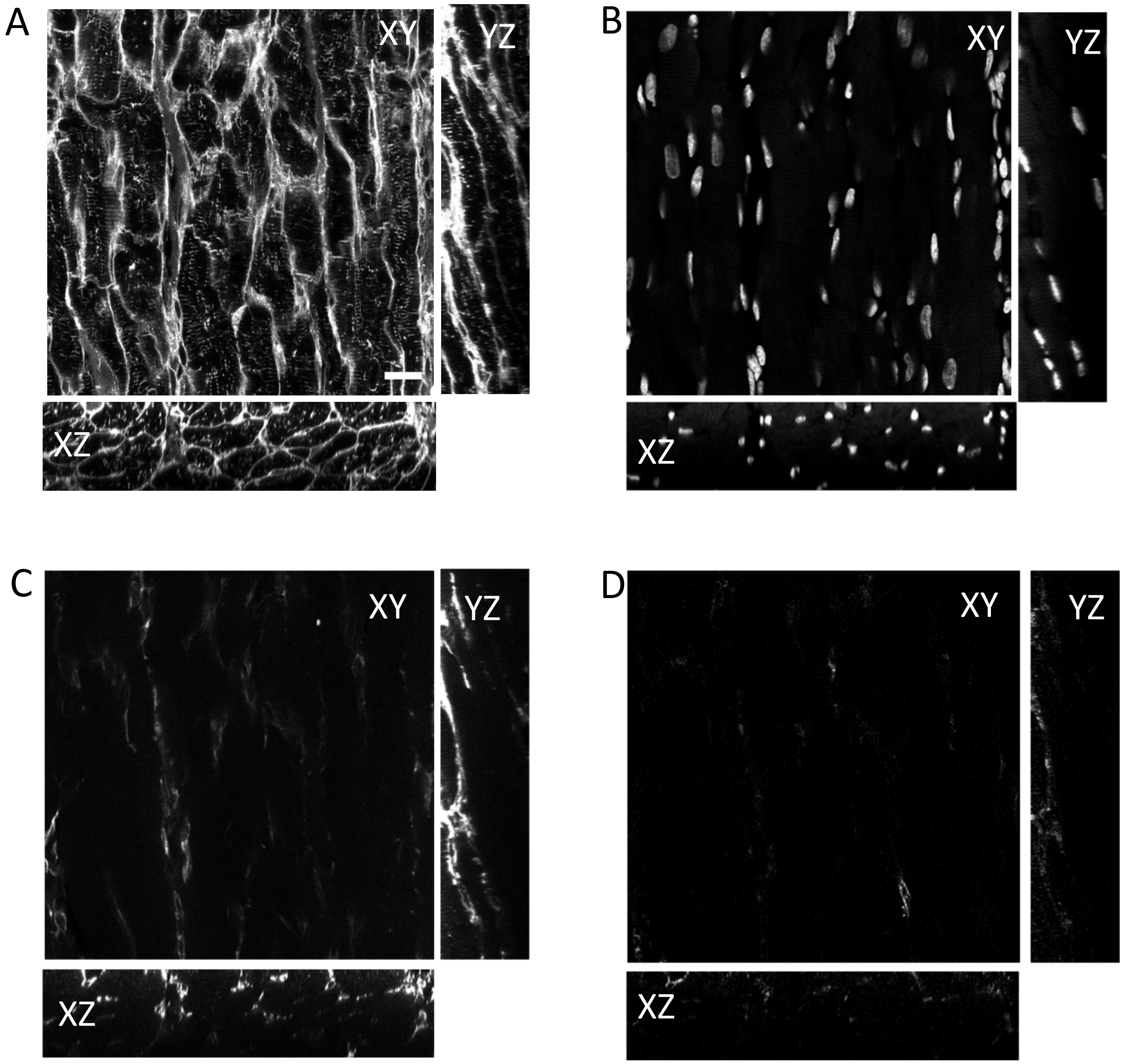

Figure S3: Raw images of central cross-sections from a 3D stack generated from region 2 of infarct tissue labeled with **(A)** WGA, **(B)** DAPI, **(C)** vimentin, and **(D)** α-SMA**.** Scale bar in **(A)** has a length 20 μm and applies to **(B-D)**.

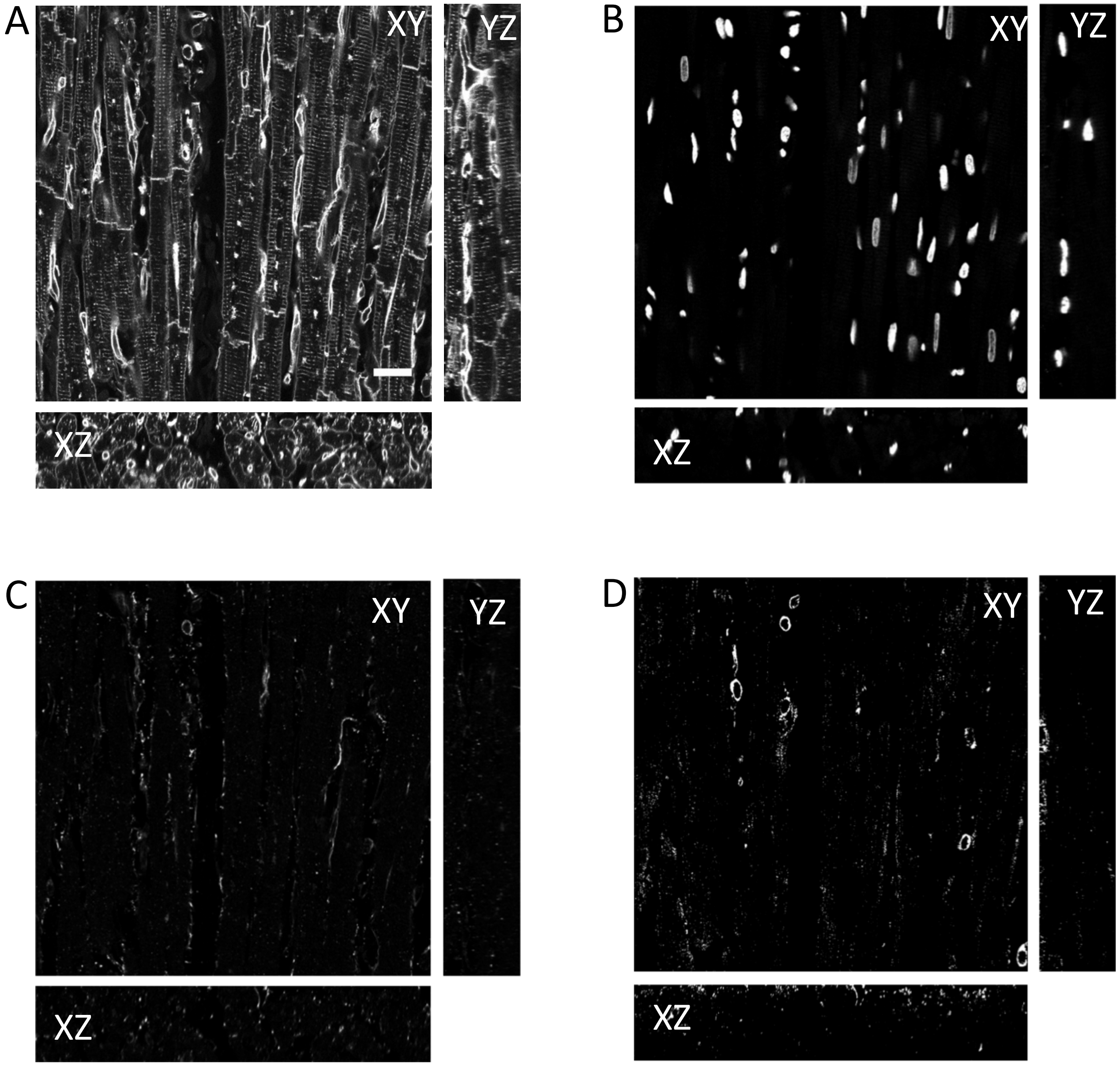

Figure S4: Raw images of central cross-sections from a 3D stack generated from region 3 of infarct tissue labeled with **(A)** WGA, **(B)** DAPI, **(C)** vimentin, and **(D)** α-SMA. Scale bar in **(A)** has a length 20 µm and applies to **(B-D)**.

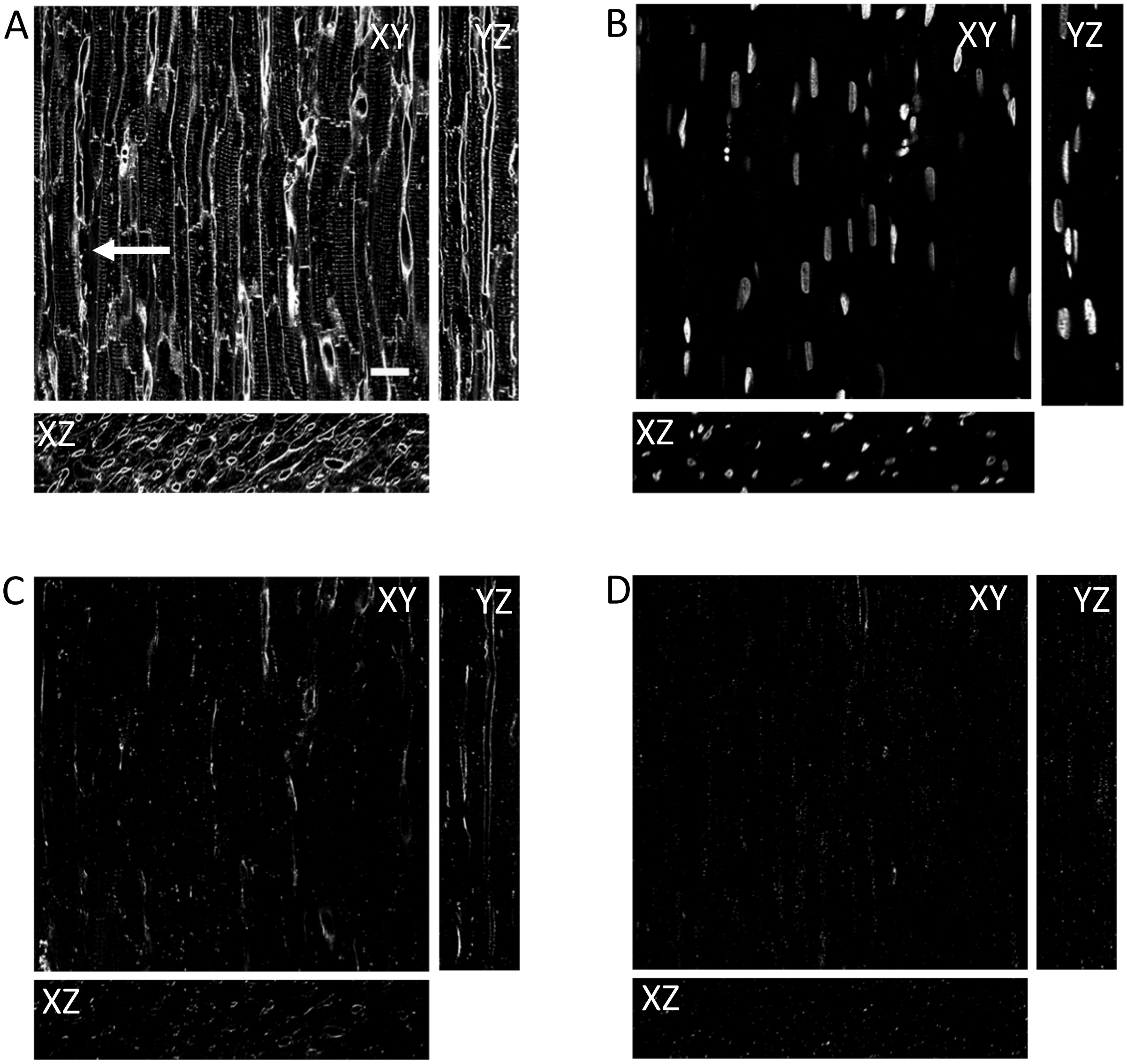

Figure S5: Images of central cross-sections after deconvolution, noise filtering and attenuation correction of a 3D image stack from control tissue labeled with **(A)** WGA, **(B)** DAPI, **(C)** vimentin, and **(D)** α-SMA. In **(A)** an interlaminar cleft is marked with an arrow. Scale bar in **(A)** has a length 20 µm and applies to **(B-D)**.

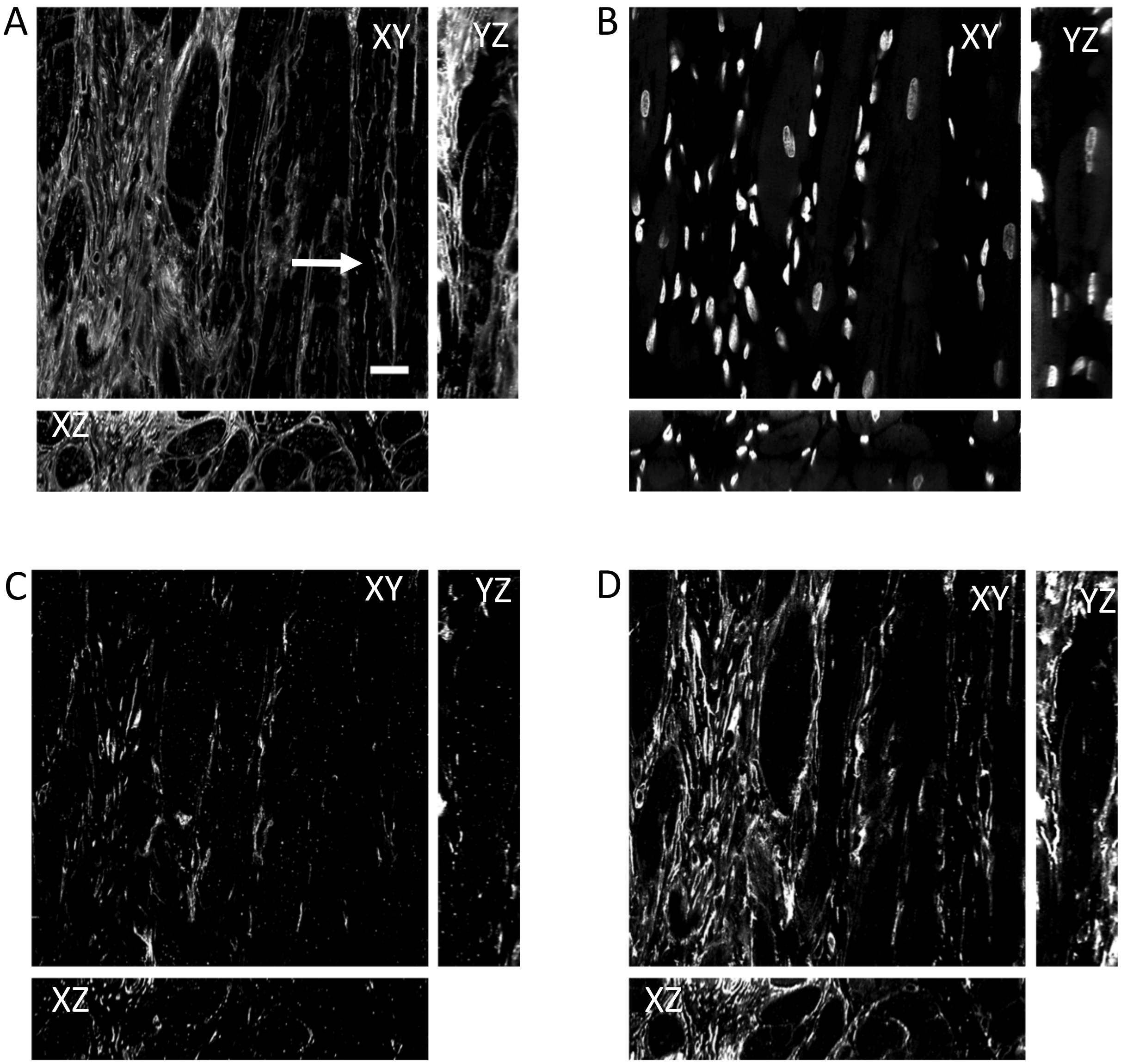

Figure S6: Images of central cross-sections after deconvolution, noise filtering and attenuation correction of a three-dimensional stack from region 1 of infarct tissue labeled with **(A)** WGA, **(B)** DAPI, **(C)** vimentin, and **(D)** α-SMA. In **(A)** an interlaminar cleft is marked with an arrow. Scale bar in **(A)** has a length 20 μm and applies to **(B-D)**.

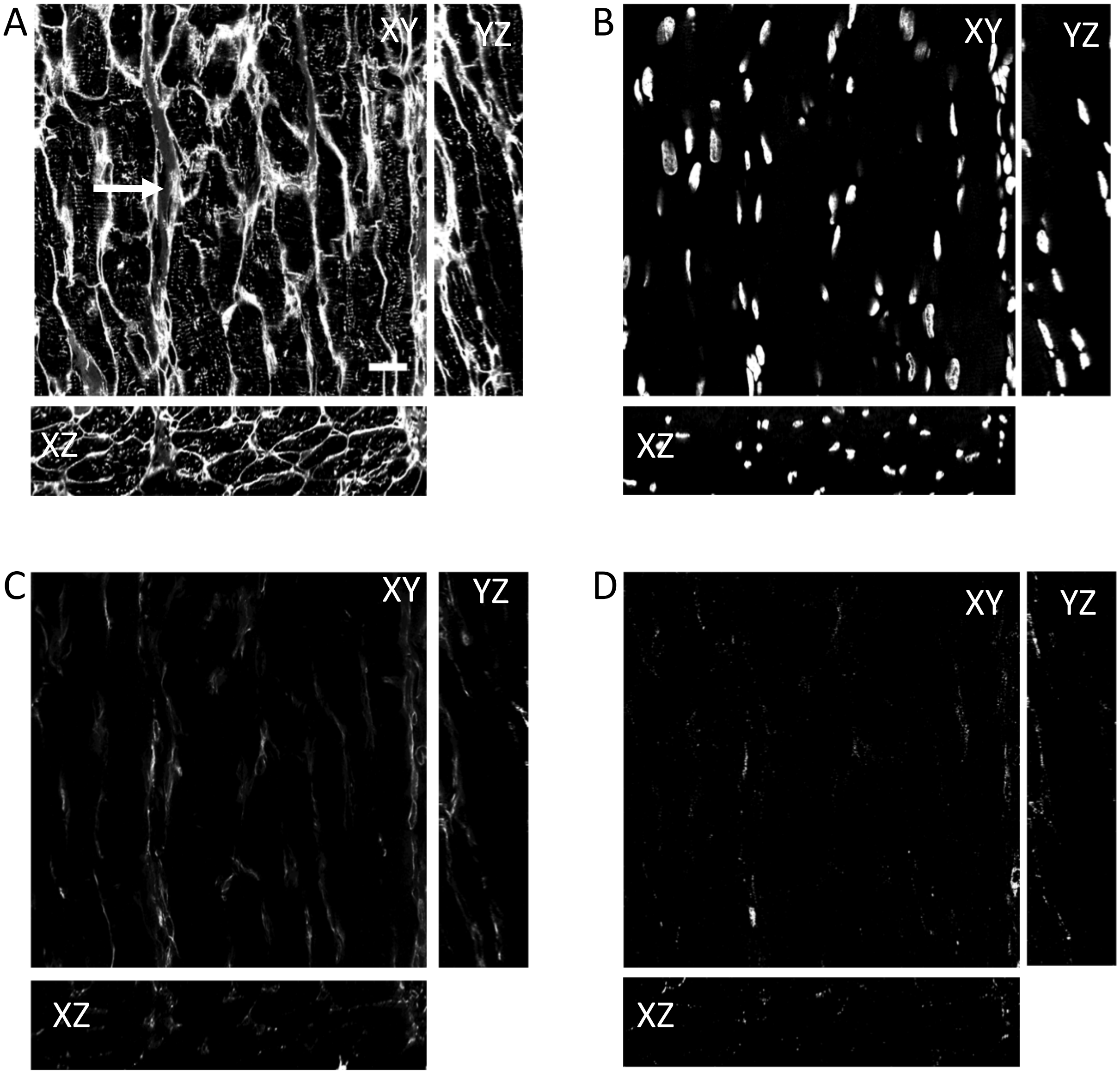

Figure S7: Images of central cross-sections after deconvolution, noise filtering and attenuation correction of a three-dimensional stack from region 2 of infarct tissue labeled with **(A)** WGA, **(B)** DAPI, **(C)** vimentin, and **(D)** α-SMA. In **(A)** an interlaminar cleft is marked with an arrow. Scale bar in **(A)** has a length 20 µm and applies to **(B-D)**.

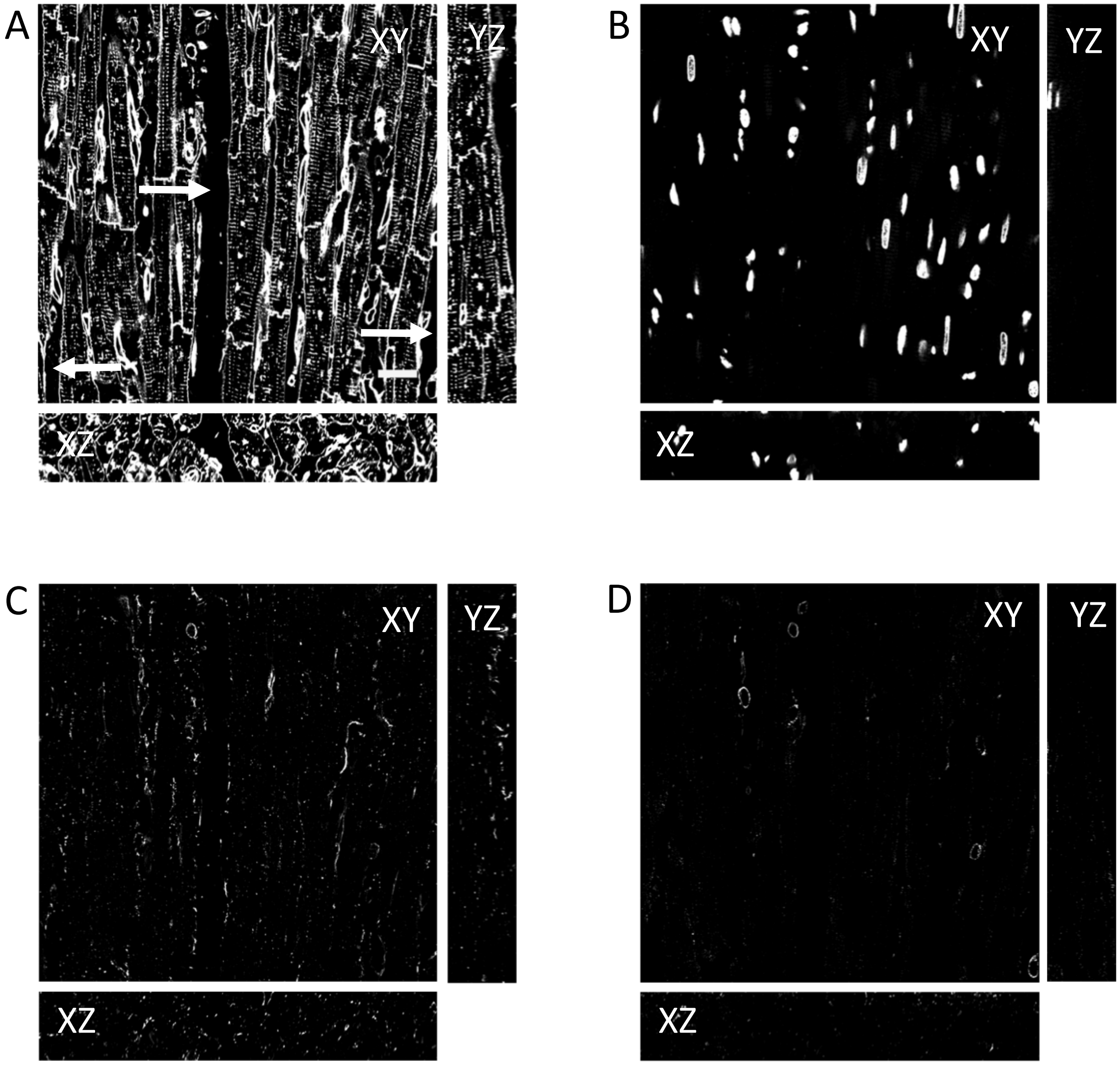

Figure S8: Images of central cross-sections after deconvolution, noise filtering and attenuation correction of a three-dimensional stack from region 3 of infarct tissue labeled with **(A)** WGA, **(B)** DAPI, **(C)** vimentin, and **(D)** α-SMA. In **(A)** interlaminar clefts are marked with arrows. Scale bar in **(A)** has a length 20 µm and applies to **(B-D)**.

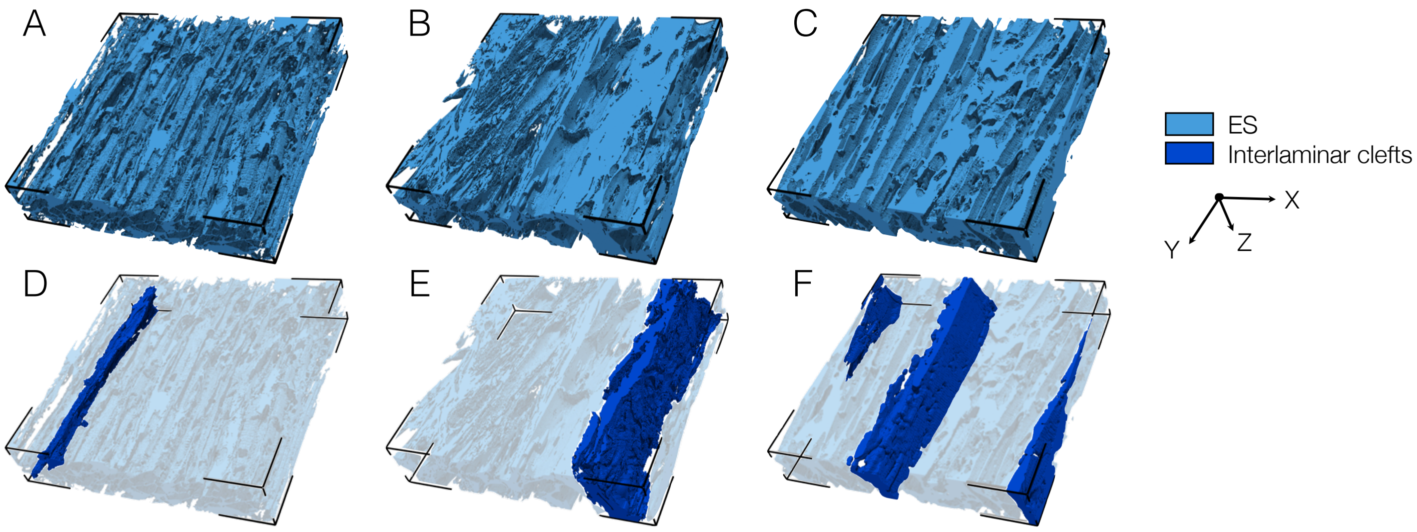

Fig. S9: 3D reconstructions of interlaminar clefts corresponding to (A) Fig. 3, (B) Fig.3 and (C) Fig. 5 for control tissue, region 1 and 3, respectively.

Table S1: List of fluorescent markers including primary and secondary antibodies used for confocal microscopic imaging

Catalog #	Name	Type	Manufacturer	Marker of
ab11369	Anti-Connexin 43 / GJA1 antibody [CXN-6]	Primary, Mouse monoclonal	abcam	Connexin 43
A21044	Goat anti-Mouse IgM (Heavy chain) Cross-Adsorbed	Secondary, Alexa Fluor 594	ThermoFischer Scientific	
A5228	Anti-Actin, α-Smooth Muscle antibody	Primary, Mouse monoclonal	Sigma Aldrich	α-Smooth Muscle Actin
A21137	Goat anti-Mouse IgG2a Cross-Adsorbed	Secondary, Alexa Fluor 555	ThermoFischer Scientific	
V6630	Anti-Vimentin antibody	Primary, Mouse monoclonal	Sigma Aldrich	Vimentin
A21240	Goat anti-Mouse IgG1 Cross-Adsorbed	Secondary, Alexa Fluor 647	ThermoFischer Scientific	
D3571	4',6-Diamidino-2-Phenylindole, Dilactate (DAPI)	Fluorescent stain (358/461 nm)	ThermoFischer Scientific	Nuclei
CF488A	CF®488A Wheat Germ Agglutinin (WGA)	Fluorescent stain (490/515 nm)	Biotium	Glycoproteins (extracellular matrix)

Table S2: Regression models showing independence between V_{cleft} and V_e-V_{cleft} and their additive nature.

Model	Variables	R^2	Slope
Linear regression	$V_{cleft} – V_e\text{-}V_{cleft}$	0.0180	-0.1833
Multiple linear regression	$\sigma_{e,l} – a{*}V_{cleft} + b{*}\,(V_e - V_{cleft})$	a=1.9382<br>b= 1.4971	0.836
	$\sigma_{e,t} – a{*}V_{cleft} + b{*}\,(V_e - V_{cleft})$	a= 2.1627<br>b= 1.8674	0.893
	$\sigma_{e,n} – a{*}V_{cleft} + b{*}\,(V_e - V_{cleft})$	a= 0.44824<br>b= 0.65529	0.665
